# Degradation mechanism of hybrid tin-based perovskite solar cells and the critical role of tin (IV) iodide

**DOI:** 10.1038/s41467-021-22864-z

**Published:** 2021-05-14

**Authors:** Luis Lanzetta, Thomas Webb, Nourdine Zibouche, Xinxing Liang, Dong Ding, Ganghong Min, Robert J. E. Westbrook, Benedetta Gaggio, Thomas J. Macdonald, M. Saiful Islam, Saif A. Haque

**Affiliations:** 1 Department of Chemistry and Centre for Processable Electronics, Molecular Sciences Research Hub, Imperial College London, London, UK; 2 Department of Chemistry, University of Bath, Bath, UK

**Keywords:** Energy, Solar cells

## Abstract

Tin perovskites have emerged as promising alternatives to toxic lead perovskites in next-generation photovoltaics, but their poor environmental stability remains an obstacle towards more competitive performances. Therefore, a full understanding of their decomposition processes is needed to address these stability issues. Herein, we elucidate the degradation mechanism of 2D/3D tin perovskite films based on (PEA)_0.2_(FA)_0.8_SnI_3_ (where PEA is phenylethylammonium and FA is formamidinium). We show that SnI_4_, a product of the oxygen-induced degradation of tin perovskite, quickly evolves into iodine via the combined action of moisture and oxygen. We identify iodine as a highly aggressive species that can further oxidise the perovskite to more SnI_4_, establishing a cyclic degradation mechanism. Perovskite stability is then observed to strongly depend on the hole transport layer chosen as the substrate, which is exploited to tackle film degradation. These key insights will enable the future design and optimisation of stable tin-based perovskite optoelectronics.

## Abstract (web summary)

Tin perovskites have emerged as promising alternatives to toxic lead perovskite in next-generation photovoltaics, but the poor environmental stability remains an obstacle for the application. Here, the authors study the degradation mechanism of tin perovskite films, and identify a cyclic degradation mechanism involving tin (IV) iodide.

## Introduction

Hybrid lead halide perovskites remain at the forefront of research activity on next-generation solar cells. Power conversion efficiencies (PCEs) for APbI_3_ perovskites (where A is typically CH_3_NH_3_^+^/MA^+^, CH_3_(NH_2_)_2_^+^/FA^+^ and/or Cs^+^) have evolved from 3.8% to 25.5% within the last decade, surpassing well-established solar cells based on polycrystalline silicon and CuInGaSe_2_^1,2^. Their outstanding performance is due to their favourable properties such as high carrier diffusion lengths, broad absorption in the visible and near infra-red, and low density of trap states^3–9^.

However, the widespread commercial scaleup of Pb perovskite devices raises concerns in relation to potential health and environmental hazards that their Pb content may cause. As such, this makes the development of Pb-free and environmentally friendly perovskite alternatives a high priority. In order to mitigate the toxicity of Pb-halide perovskites but simultaneously retain their favourable photovoltaic properties, Pb^2+^ can be replaced by lower-toxicity cations with similar outer shell electron configurations, such as Sn^2+^, Ge^2+^, Bi^3+^ or Sb^3+^^10–15^. Among these options, Sn halide perovskites have emerged as the most promising alternative^16–29^, exhibiting significantly lower bioavailability compared to Pb-based perovskites^30^ and delivering the highest PCEs among Pb-free perovskite solar cells since devices based on CH_3_NH_3_SnI_3−*x*_Br_*x*_ were reported in 2014^10,11^. Interest in these materials also arises from their superior semiconductor properties compared to their Pb analogues, such as broader absorption range, nearly-ideal bandgaps (~1.3 eV) and higher charge carrier mobilities^31,32^.

With record PCEs surpassing 13%^33^, Sn-based perovskite solar cells have steadily become more competitive due to intensive research efforts. However, the device performance of tin perovskite solar cells has advanced at a slower pace relative to their Pb counterparts, mainly due to their poorer stability under ambient environmental conditions. Such stability issues with these materials relate mainly to the facile oxidation of Sn^2+^ to Sn^4+^, which is also known to introduce p-type self-doping in the perovskite^34^. This in turn leads to high rates of monomolecular electron-hole recombination and therefore poor solar cell performance^35^. A number of strategies have been explored to address these issues, which include the use of SnX_2_ additives to mitigate self-doping^36,37^, as well as the introduction of inherently more stable low-dimensional phases^38–46^. However, these approaches do not completely solve the problem and therefore a full elucidation of the decomposition pathways of Sn perovskites is needed to address the stability bottleneck more effectively.

To date, reports describing the degradation mechanism of Sn perovskites remain limited, in contrast with Pb-based analogues^47–53^. For example, some studies have shown that ASnX_3_ (A = Cs^+^, MA^+^ or FA^+^; X = I^-^, Br^-^) can decompose in air to form A_2_SnX_6_ (vacancy-ordered double perovskite^54^) and SnO_2_^31,55–57^_,_ while others revealed that FASnI_3_ and (PEA)_2_SnI_4_ (where PEA refers to phenylethylammonium) degrade in the presence of oxygen to SnI_4_, SnO_2_ and FAI/PEAI^58,59^. Whilst these findings provide plausible air-mediated decomposition routes of Sn-based perovskites, a more detailed understanding of the degradation mechanism as well as knowledge of inconspicuous reaction pathways is required. For example, it is reasonable to suppose that SnI_4_ can participate in further degradation reactions of ASnI_3_ on account of its high reactivity with water and oxygen relative to FAI, SnO_2_ and A_2_SnI_6_^11,60–65^. Therefore, a more in-depth understanding of the role of SnI_4_ in tin perovskite degradation is necessary.

In this paper, we report on the degradation mechanism of ASnI_3_ perovskites (where A represents 20% PEA and 80% FA) under ambient environmental conditions using a combination of diffraction, spectroscopy and ab initio simulation techniques. Herein, we identify SnI_4_ as a major contributor to the degradation process and therefore to the instability of such perovskites. Specifically, SnI_4_ is a direct product of the decomposition of 2D/3D (PEA)_0.2_(FA)_0.8_SnI_3_ under ambient air^58,59^. We go on to explore the impact of SnI_4_ on the optoelectronic properties, solar cell performance and stability of (PEA)_0.2_(FA)_0.8_SnI_3_ films. We show that the presence of SnI_4_ leads to enhanced non-radiative recombination and poorer device PCE, which is due to high free hole density caused by the introduced Sn^4+^ states. Crucially, we observe that SnI_4_-richer perovskite films degrade faster under ambient conditions, suggesting that SnI_4_ accelerates the decomposition of Sn-based perovskites upon exposure to ambient air.

**Fig. 1:**
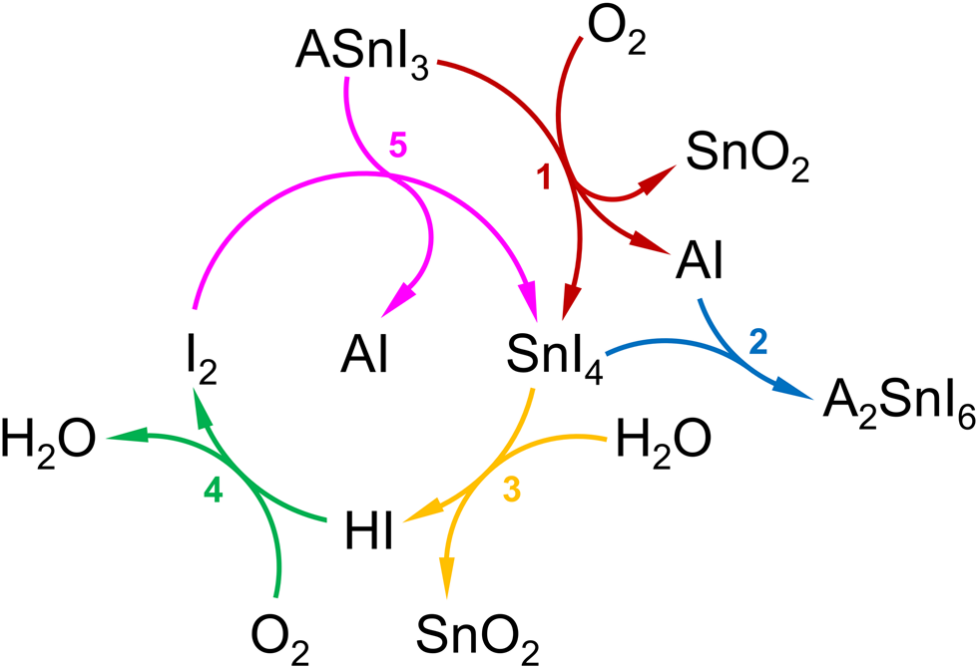
Proposed cyclic degradation mechanism of a tin iodide perovskite under ambient air exposure. Unless stated otherwise, ‘A’ refers to the organic cations chosen for the preparation of the hybrid tin perovskite (20% PEA and 80% FA in the present work). Reaction 1: oxidation of perovskite by O_2_ and SnI_4_ formation; Reaction 2: solid-state formation of A_2_SnI_6_ from SnI_4_ and AI, where A ≡ FA. Reaction 3: hydrolysis of SnI_4_ by H_2_O and HI formation; Reaction 4: oxidation of HI by O_2_ and I_2_ formation; Reaction 5: oxidation of perovskite by I_2_ and SnI_4_ formation.

We then investigate the role of SnI_4_ in the degradation mechanism of (PEA)_0.2_(FA)_0.8_SnI_3_ films and demonstrate that SnI_4_ in the film readily evolves to form I_2_ via a two-step process, namely, (i) the hydrolysis reaction of SnI_4_ with H_2_O to give HI and (ii) the oxidation of HI by O_2_ to form I_2_. Interestingly, the perovskite is found to rapidly degrade when exposed to I_2_ resulting in the formation of more SnI_4_, thus establishing a cyclic degradation mechanism shown schematically in Fig. 1.

Finally, we find that the stability of (PEA)_0.2_(FA)_0.8_SnI_3_ perovskite films are highly dependent on the hole transport layer chosen as a substrate (i.e., NiO_*x*_, CuSCN and PEDOT:PSS). We observe an improvement in perovskite film stability as the hole withdrawal ability of the bottom layer is increased. We suggest that this process chemically reduces the perovskite film, mitigating the high sensitivity of the Sn-based perovskite to both exogenous and endogenous oxidising species (O_2_ and I_2_). Thus, we expect the implementation of highly efficient hole acceptors to provide a pathway towards more stable tin-based perovskite optoelectronics.

## Results

### Degradation in air

**Fig. 2:**
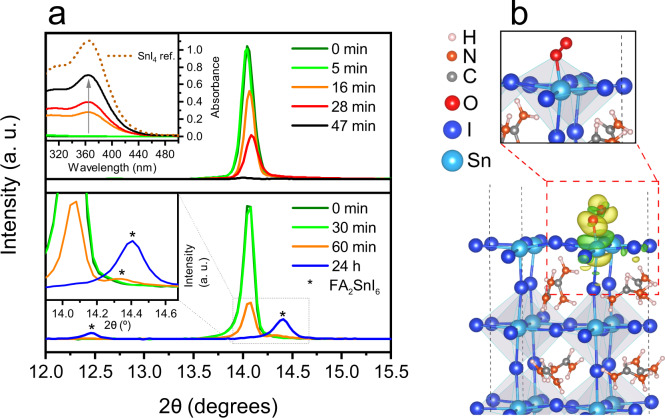
Degradation of Sn perovskite under ambient air. **a** Time evolution of the (100) XRD reflection of (PEA)_0.2_(FA)_0.8_SnI_3_ films exposed to ambient air at 100 °C (top; inset: absorbance spectra of SnI_4_ dissolved in 3 mL of toluene after its sublimation from the heated film and capture in a Petri dish during different times) and room temperature (bottom; inset: magnification of peaks corresponding to 60 min and 24 h). Peaks labelled with * correspond to FA_2_SnI_6_. **b** Adsorption of an O_2_ molecule on a (001) surface of FASnI_3_ perovskite and charge density difference, which highlights bond formation between O and Sn atoms (from DFT simulations). Yellow and green charge densities refer to electron accumulation and depletion respectively (with an isosurface value of 0.006 Å^−3^).

As can be seen in Fig. 1, SnI_4_ plays a pivotal role in the degradation of tin iodide perovskites. As such, we first identify the presence of this species as a degradation product of 2D/3D (PEA)_0.2_(FA)_0.8_SnI_3_ thin films comparable to those employed in state-of-art solar cells^39–42,44^. Unless specified otherwise, all degradation experiments reported herein were carried out by exposing samples to ambient air (relative humidity, RH = 38.0 ± 7.5%; temperature, T = 22.9 ± 0.9 °C), consistent with normal working conditions. Figure 2a shows the time evolution of the (100) reflection (orthorhombic system, *Amm2* space group; full pattern in Supplementary Fig. 5a) obtained from the X-ray diffraction (XRD) patterns of (PEA)_0.2_(FA)_0.8_SnI_3_ films aged at 100 °C (Fig. 2a, top) and room temperature (Fig. 2a, bottom).

Tin perovskite films degraded under ambient conditions whilst subjected to 100 °C heating show a rapid drop in the (100) reflection as they evolve in appearance from black/brown to transparent. This observation is consistent with the disruption of the perovskite crystalline structure due to the oxidative effect of atmospheric oxygen. Such degradation results in the formation of SnI_4_, which rapidly sublimes under heating. Evidence for the sublimation of SnI_4_ was obtained by covering the perovskite films with a glass Petri dish during the experiment and immediately extracting the captured products with toluene. The UV–Visible spectra of the captured product (Fig. 2a, top (inset)) clearly indicate the presence of SnI_4_ as a degradation product. We, therefore, propose Reaction 1 as the first step of Sn perovskite degradation in air, in good agreement with previous reports^58,59^:

1 $$2\,{{\rm{ASnI}}}_{3}+{{\rm{O}}}_{2}\to {{\rm{SnI}}}_{4}+{{\rm{SnO}}}_{2}+2\,{\rm{AI}}$$

To further complement the experimentally proposed degradation pathways of the Sn perovskite film, we have examined the energetics of Reaction 1 for FASnI_3_ using density functional theory (DFT) methods. We note that the FA perovskite system was simulated, rather than including PEA, due to the lack of precise experimental crystal structural data for PEA/FA systems similar to the (PEA)_0.2_(FA)_0.8_SnI_3_ precursor ratio as a reliable starting point and to allow a more focused approach on deriving trends of the reaction and surface properties. The reaction energy is calculated as the difference between the products and the reactants (in which a negative value indicates a favourable exothermic reaction). Details of the simulations and procedures, which have been applied recently to tin halide perovskites^66,67^, are provided in the Methods section. We calculate a highly favourable energy (−3.46 eV) for Reaction 1, which indicates that the perovskite film indeed degrades in the presence of oxygen as the initial step to form FAI, SnO_2_, and SnI_4_ in accord with the experimentally observed reaction products.

To examine the microscopic mechanisms of this reaction with oxygen, we also simulated the adsorption of an O_2_ molecule on the FASnI_3_ perovskite surface. We examined three possible adsorption configurations of an O_2_ molecule on the (001) surface with FA/I and Sn/I terminations. We focused on the (001) surface as it has been shown to be one of the most stable and most extensively studied halide perovskite surfaces^68,69^. In the case of FA/I termination, the O_2_ molecule remains at a certain distance from the surface after relaxation independent of the initial configuration showing no interaction with surface species, which indicates that adsorption does not take place (as shown in Supplementary Fig. 22). For the Sn/I terminated surface, the most stable adsorption structure after relaxation is shown in Fig. 2b, which corresponds to the initial configuration of O_2_ located right above an Sn atom (for additional views, see Supplementary Fig. 18b). It is clear that one of the oxygen atoms of the O_2_ molecule forms a chemical bond with the Sn atom at the Sn/I terminated surface, as shown by the charge density difference in Fig. 2b.

Regarding the thermodynamics of adsorption on the surface, we have calculated an adsorption energy of 0.7 eV. The Sn–O bond length is found to be 2.31 Å, which is close to the experimental Sn–O bond length in SnO_2_ (~2.1 Å^70^). We also found that the O–O distance is stretched by over 0.1 Å beyond the bond length of the O_2_ molecule; this elongation suggests that the O_2_ molecule is likely to be dissociated on the surface and may subsequently participate in the formation of SnO_2_ (which is a phase detected in our samples via X-ray photoelectron spectroscopy (XPS); Supplementary Fig. 7 and Supplementary Table 1). Our simulation results are therefore consistent with the experimentally proposed Reaction 1 and provide additional atomistic insights into the reaction mechanism of the initial stage in the oxidation of the tin perovskite.

The XRD data in Fig. 2a (bottom panel) show that in the film degraded at room temperature the (100) peak intensity also decreases during the course of an hour as a result of ambient air mediated degradation (relative signal loss vs time: 0% at 0 min, 3% at 30 min, 73% at 60 min; see Supplementary Fig. 1a). In addition, we observe the appearance of a small diffraction peak at 2*θ* ~14.4° (see Fig. 2a, bottom (inset), 60 min), which we attribute to the vacancy-ordered double perovskite variant FA_2_SnI_6_^71^ (see a full pattern after 24 h of exposure to ambient air in Supplementary Fig. 1b). However, the emergence of this phase appears to be delayed as compared to perovskite degradation (12% rise of FA_2_SnI_6_ peak vs. 73% drop of (PEA)_0.2_(FA)_0.8_SnI_3_ peak after 60 min; Supplementary Fig. 1a). This suggests that degradation products formed via Reaction 1 gradually accumulate in the film for a prolonged time (~30–60 min) before triggering the solid-state process between SnI_4_ and FAI to form FA_2_SnI_6_ (Reaction 2)^71,72^:

2 $${{\rm{SnI}}}_{4}+2\,{\rm{AI}}\to {{\rm{A}}}_{2}{{\rm{SnI}}}_{6}$$

Taken together, the data in Fig. 2 suggest that the presence of FA_2_SnI_6_ in the film only occurs after substantial perovskite degradation and therefore the early interaction between this phase and (PEA)_0.2_(FA)_0.8_SnI_3_ is unlikely. In contrast, it is evident that SnI_4_, SnO_2_, FAI and PEAI coexist with the tin perovskite in the film during the initial stages of the material decomposition. DFT simulations indicate a very small energy (−0.21 eV) for Reaction 2 involving SnI_4_ interacting with FAI to form the FA_2_SnI_6_ phase, which is consistent with the slower formation of this phase. Upon degrading the sample under heating, we also note that the eventual formation of FA_2_SnI_6_ is impeded due to the rapid sublimation of SnI_4_.

### Optoelectronic and stability effects of tin (IV) iodide

Next, we examine the effect of SnI_4_ on the optoelectronic properties, device performance and ambient stability of (PEA)_0.2_(FA)_0.8_SnI_3_ films. For this, the SnI_4_ content in the perovskite films was varied by tuning the SnI_4_ concentration in the perovskite precursor solution by both thermally purifying the control (commercially sourced) SnI_2_ precursor to minimise its SnI_4_ impurities and by replacing 2 mol% control SnI_2_ by equimolar amounts of SnI_4_. We note that details of precursor purification (experimental setup and conditions, Supplementary Fig. 2; thermogravimetric analysis (TGA), Supplementary Fig. 3a; SnI_4_ identification and quantification, Supplementary Fig. 4), the effect of control/purified SnI_2_ on the perovskite film structure (XRD, Supplementary Fig. 5), morphology (scanning electron microscopy (SEM), Supplementary Fig. 6), and composition (XPS; Supplementary Fig. 7 and Supplementary Table 1) are provided in the Supplementary Information.

**Fig. 3:**
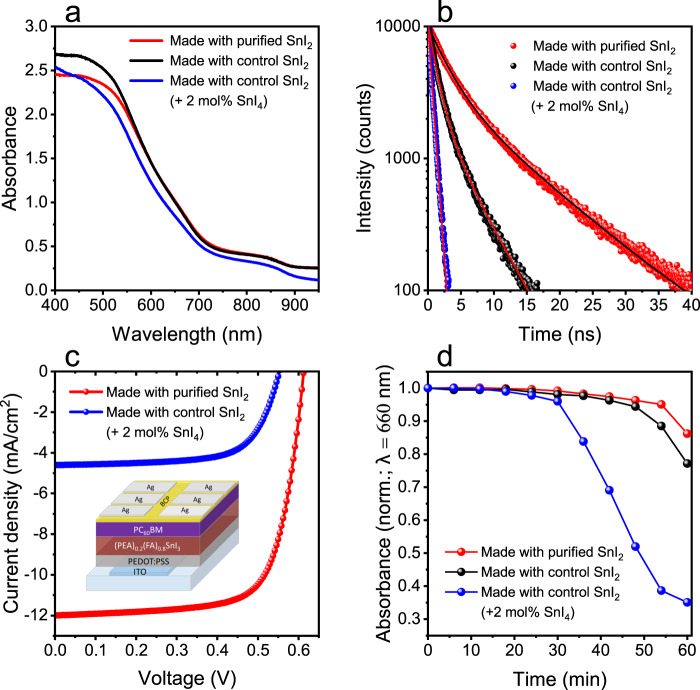
Effect of tin (IV) iodide on Sn perovskite optical properties, solar cell performance and stability. **a** UV–Visible spectra of glass/(PEA)_0.2_(FA)_0.8_SnI_3_ thin films made with varying SnI_4_ concentrations. **b** Time-resolved PL decays of perovskite films acquired at 875 nm with 404 nm excitation and their fitting curves. Average *τ*_*f*_ for films are 5.49 ns (film made with purified SnI_2_; biexponential decay fit), 2.09 ns (film made with control SnI_2_; biexponential decay fit) and 0.60 ns (film with added SnI_4_; monoexponential decay fit). **c**. Current density–voltage (J–V) curves of champion solar cells based on (PEA)_0.2_(FA)_0.8_SnI_3_ made with varying SnI_4_ concentrations. **d**. Time evolution of relative absorbance at 660 nm of perovskite thin films made with varying SnI_4_ concentrations. Degradation experiments were carried out under dark and ambient air conditions (23 °C, 38% RH).

Figure 3a shows the UV–Visible absorption characteristics of three perovskite films: sample (A), film made with purified commercial SnI_2_ (0.06% SnI_4_ content); sample (B), film made with as-received commercial SnI_2_ (0.65% SnI_4_ content) and sample (C), film made with commercial SnI_2_ and 2 mol% SnI_4_ (3.95% SnI_4_ content). We note that XPS measurements confirm an increase in Sn^4+^ content in films processed with SnI_4_-richer SnI_2_ precursors (Supplementary Fig. 7). Moreover, it is apparent from Fig. 3a that the optical bandgaps of samples (A) and (B) are found to be ~1.32 eV, while that of sample (C) is 1.35 eV (Tauc plots provided in Supplementary Fig. 8). It is possible that the larger bandgap observed in sample (C) may originate from its more pronounced p-type character; a higher SnI_4_ content introduces more Sn^4+^ states in the perovskite, resulting in a hole-richer valence band and therefore causing band-to-band electron transitions to occur at higher energies.

Figure 3b presents time-resolved photoluminescence (PL) decays for samples (A), (B) and (C). It is apparent that longer fluorescence lifetimes are observed in films made with less exogenous SnI_4_ (sample A: *τ* = 5.49 ns > sample B: *τ* = 2.09 ns > sample C: τ = 0.60 ns). We attribute the increase in emission lifetime in sample A as compared to samples B and C to a reduction in non-radiative monomolecular recombination rates that arise from the fewer Sn^4+^ states that are incorporated into the perovskite structure; this being in good agreement with our XPS data (Supplementary Fig. 7 and Supplementary Table 1) and previous work^73^. Taken together, the data presented in Fig. 3a, b highlight the detrimental impact of SnI_4_ on the photophysical properties of Sn-based perovskites. It is pertinent to note that purifying the precursor (via sublimation) prior to film processing results in an improvement in the optoelectronic properties of the material.

We next consider the influence of SnI_4_ on device performance. Figure 3c shows current density–voltage (J–V) curves of Sn perovskite solar cells based on a typical inverted architecture (ITO/PEDOT:PSS/(PEA)_0.2_(FA)_0.8_SnI_3_/PC_60_BM/BCP/Ag; see inset). Photovoltaic figures of merit of our devices are summarised in Supplementary Fig. 9 (box plots) and Supplementary Table 2. Solar cells made with the purified SnI_2_ attain high efficiencies (5.23%) compared to previous reports based on similar 2D/3D absorbers^39,74^. The high open-circuit voltage (*V*_*oc*_) (0.61 V) and fill factor (71%) achieved indicate low trap-mediated recombination and balanced charge transport. However, the relatively low short-circuit current density (*J*_*sc*_) is possibly caused by charge losses at perovskite/PEDOT:PSS and/or perovskite/PC_60_BM interfaces. In contrast, it is evident from J–V data in Fig. 3c that increasing the SnI_4_ content in the perovskite layer leads to a significant drop in the PCE (5.23% > 1.73%) due to a drop in *V*_*oc*_ (0.61 V > 0.55 V) and *J*_*s*c_ (12.00 mA/cm^2^ > 4.61 mA/cm^2^); this is consistent with increased carrier recombination and lower carrier mobility as Sn^4+^ doping is introduced^75,76^.

Next, we consider the effect of SnI_4_ on the stability of (PEA)_0.2_(FA)_0.8_SnI_3_ films. Contour graphs showing normalised absorbance of samples (A), (B) and (C) as a function of time and wavelength are shown in Supplementary Fig. 10. These measurements indicate a drop in absorbance across the spectrum but more notably around 660 and 870 nm, close to the energies of the two main allowed optical transitions in these materials^77^. Normalised absorbance decays at 660 nm are plotted in Fig. 3d for comparison. The data presented in Fig. 3d show that the presence of SnI_4_ significantly increases the rate of degradation of (PEA)_0.2_(FA)_0.8_SnI_3_ films (stability: sample (A) > sample (B) > sample (C)).

### Evolution of tin (IV) iodide to iodine

So far, we have shown that: (i) SnI_4_ is generated as a product of the oxygen-induced degradation of Sn-based perovskite films and (ii) the presence of SnI_4_ in the film is highly detrimental to the optoelectronic properties, device performance and ambient stability of (PEA)_0.2_(FA)_0.8_SnI_3_ films. Whilst the deterioration of the optoelectronic properties and photovoltaic performance in the material can be attributed to non-radiative recombination caused by Sn^4+^ dopants, the role of SnI_4_ in the degradation mechanism and stability of Sn perovskites remains unclear. It is reasonable to suppose that SnI_4_ can spontaneously evolve into iodine in the presence of moisture and oxygen^11,60–65^ as indicated in Fig. 1. This is in agreement with empirical thermodynamic data^78,79^ showing that (i) SnI_4_ can hydrolyse to form SnO_2_ and HI (Gibbs free energy: Δ*G*° = −0.60 eV) and, similarly, (ii) HI can react with oxygen to give I_2_ and H_2_O (Δ*G*° = −2.20 eV). To investigate whether these reactions are feasible in the perovskites reported herein, a combination of proton nuclear magnetic resonance (^1^H-NMR) and UV–Visible spectroscopies were used.

**Fig. 4:**
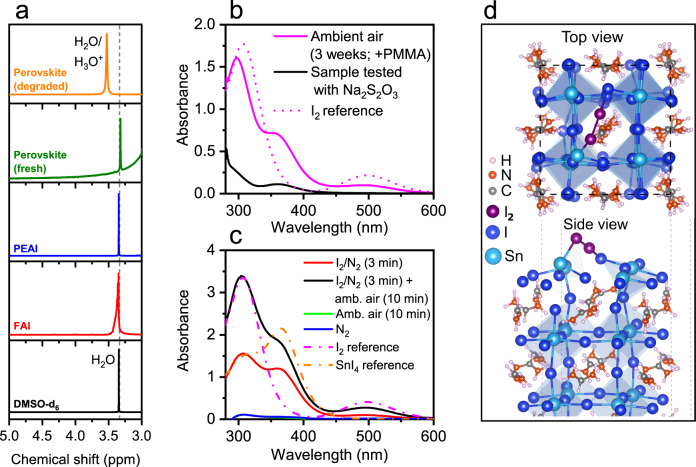
Evolution of tin (IV) iodide to iodine and Sn perovskite oxidation by iodine. **a** Upfield ^1^H-NMR spectra of DMSO-d_6_, FAI, PEAI, fresh (PEA)_0.2_(FA)_0.8_SnI_3_ and (PEA)_0.2_(FA)_0.8_SnI_3_ aged for 24 h in ambient air. **b** UV–Visible spectra of degradation products extracted in toluene (3 mL) from a PMMA-coated (PEA)_0.2_(FA)_0.8_SnI_3_ thin-film fully aged in ambient air for 3 weeks, the same solution after Na_2_S_2_O_3_ addition and I_2_ reference solution (dotted line) **c** UV–Visible spectra of degradation products extracted in toluene (3 mL) from (PEA)_0.2_(FA)_0.8_SnI_3_ thin films aged under different conditions (indicated in legend) and SnI_4_ and I_2_ reference solutions (dashed lines). **d** Top and side views of the structural arrangement of an adsorbed I_2_ molecule (purple) on the (001) surface of FASnI_3_ (from DFT calculations).

First, ^1^H-NMR spectroscopy is used to analyse the interaction of SnI_4_ with moisture in a (PEA)_0.2_(FA)_0.8_SnI_3_ film degraded under ambient air for 24 h and dissolved in deuterated DMSO (Fig. 4a). We note that full (downfield) NMR spectra of the solvent, FAI, PEAI, fresh (PEA)_0.2_(FA)_0.8_SnI_3_, aged (PEA)_0.2_(FA)_0.8_SnI_3_ and their peak assignment are provided in Supplementary Fig. 11, indicating that FA^+^ and PEA^+^ do not undergo any structural alterations within the 24 h degradation period. However, the peak assigned to H_2_O traces is detected at ~3.53 ppm in the upfield degraded perovskite spectrum, in contrast with its position at ~3.35 ppm in the reference DMSO-d_6_, FAI, PEAI and fresh perovskite spectra (Fig. 4a). The downfield shift in the H_2_O peak is indicative of a decrease in electron density around water protons (de-shielding effect), consistent with the protonation of water (i.e., formation of H_3_O^+^). The acidification of water is consistent with the formation of HI through the reaction of SnI_4_ with H_2_O, as detailed in Reaction 3^8,60,61,63–65^:

3 $${{\rm{SnI}}}_{4}+2\,{{\rm{H}}}_{2}{\rm{O}}\to 4\,{{\rm{HI}}+{\rm{SnO}}}_{2}$$

It is pertinent to note that HI can participate in further reactions under ambient conditions due to its highly reactive nature. For example, HI formed as a result of water-mediated degradation of CH_3_NH_3_PbI_3_^80,81^ has been suggested to evolve in the air to form I_2_^82^. As such, a plausible reaction route for HI oxidation to give I_2_ and H_2_O is represented in Reaction 4:

4 $$4\,{{\rm{HI}}+{\rm{O}}}_{2}\to 2\,{{\rm{I}}}_{2}+2\,{{\rm{H}}}_{2}{\rm{O}}$$

Further evidence for Reactions 3 and 4 occuring was obtained from studying the reaction of water with SnI_4_, followed by exposure to air (Supplementary Fig. 12). Specifically, upon the addition of SnI_4_ to water, we observe a drop in pH and the precipitation of a white solid, consistent with the formation of HI and SnO_2_, respectively. Moreover, ageing this mixture in air leads to a colour change (from clear to yellow/orange) along with new absorption features that indicate the oxidation of HI to I_2_ (see Supplementary Fig. 12). We note that the photoinduced decomposition of HI into its elements (H_2_ and I_2_) cannot be excluded, although we expect this path to be less favourable due to thermodynamic limitations^83–85^. Taken together, Reactions 3 and 4 suggest that SnI_4_ is highly likely to evolve into I_2_ through the cooperative action of atmospheric H_2_O and O_2_, with HI most likely acting as a short-lived, intermediate species. We note that the direct reaction of SnI_4_ with O_2_ to form I_2_ and SnO_2_ is also a possibility; nonetheless, this process is known to require high temperatures to occur^62^ and therefore we consider it to be much slower relative to Reactions 3 and 4 combined.

In order to verify the evolution of SnI_4_ into I_2_ in (PEA)_0.2_(FA)_0.8_SnI_3_ perovskite films under air, we next analyse degraded samples via UV–Visible spectroscopy to detect the halogen. The absorbance spectrum of degradation products extracted via toluene from a film aged in ambient air for 3 weeks is presented in Fig. 4b. The resulting UV–Visible characteristics in Fig. 4b show a shoulder at ~360 nm, assigned to SnI_4_, and two further features at ~300 and ~500 nm consistent with the presence of I_2_ (see I_2_ reference spectrum, Fig. 4b). The elimination of these two features by testing the solution with Na_2_S_2_O_3_, which is known to reduce I_2_ to I^−^, further supports the presence of I_2_ as a product of the degradation of SnI_4_. We note that the evolution of SnI_4_ to I_2_ is also observed in toluene solutions (Supplementary Fig. 13).

### Perovskite oxidation by iodine

The next question that arises relates to whether iodine can directly react with the perovskite and cause further degradation of the material. To investigate this, we exposed fresh (PEA)_0.2_(FA)_0.8_SnI_3_ films to artificially generated I_2_ atmospheres for short periods of time (~350 ppm I_2_, ~3 min) and used UV–Visible spectroscopy to study the reaction. Details of the experimental procedure and sample characterisation can be found in ‘Methods’. We find that Sn perovskite films exposed to I_2_ vapour result in a dramatic colour change (from black/brown to pale orange, Supplementary Fig. 14) attributed to perovskite degradation. Figure 4c shows the absorbance spectrum of degradation products extracted in toluene from an I_2_-exposed film. The absorption peak at ~360 nm reveals once more the presence of SnI_4_, confirming that I_2_ oxidises the Sn perovskite to SnI_4_ (via Reaction 5):

5 $${{\rm{ASnI}}}_{3}{+{\rm{I}}}_{2}\to {{\rm{SnI}}}_{4}+{\rm{AI}}$$

DFT calculations find a favourable energy (−0.61 eV) for this reaction (5) involving I_2_ interacting with FASnI_3_ to form SnI_4_ and FAI, which again agrees with experimental observation. To further analyse the detrimental role of I_2_, we also simulated the absorption of an I_2_ molecule on the (001) surface of FASnI_3_ in a similar methodology to the case of O_2_. Figure 4d shows the most stable structure after relaxation on the Sn/I terminated surface, which corresponds to I_2_ above the FA molecular cation (for further views, see Supplementary Fig. 20). The top view in Fig. 4d shows that the I_2_ molecule forms bridge-like bonds between the Sn and I atoms at the Sn/I terminated surface. The side view shows that the Sn atom that is bonded with one of the I atoms of the molecule is pulled out of the SnI_2_ surface, distorting the local regular Sn coordination of the perovskite structure. This suggests that the I_2_ species reacts with the perovskite crystal and most likely results in the formation of additional SnI_4_ as proposed experimentally in Reaction 5. When the surface is FA/I terminated, the I_2_ molecule interacts with the outer iodine atom of the surface and forms a triiodide-like molecular shape, independent of the initial configurations, as shown in Supplementary Fig. 23. Overall, these results show that our simulations are in line with the experimental findings of the cyclic degradation process, as well as providing new mechanistic insights at the atomic level.

### Cyclic degradation mechanism of Sn perovskite

Our findings provide a detailed description of the degradation processes in Sn perovskite films under ambient air, as schematically depicted in Fig. 1. The decomposition of the material is triggered by oxygen, which oxidises the perovskite to SnI_4_ (Reaction 1). Next, this degradation product can follow two reaction pathways, namely (i) its solid-state evolution to form a vacancy-ordered Sn(IV) double perovskite (Reaction 2) and, more critically, (ii) its evolution in the presence of moisture and oxygen via, most likely, a HI intermediate to give I_2_ (Reactions 3 and 4), which we find to be a fast process (<10 min; Supplementary Note 1). (PEA)_0.2_(FA)_0.8_SnI_3_ films are found to degrade rapidly (<3 min) when exposed to I_2_ at lower concentrations than atmospheric O_2_ (~0.035% I_2_ < ~21% O_2_). As can be seen in Fig. 1, SnI_4_ and I_2_ can interconvert while causing the decomposition of the perovskite. We believe that our mechanistic insights explain why SnI_4_ impurities in the perovskite precursor lead to greater premature perovskite degradation (Fig. 3d): the presence of exogenous SnI_4_ in the perovskite may speed up the formation of highly aggressive I_2_, which in turns leads to the degradation of the perovskite.

**Fig. 5:**
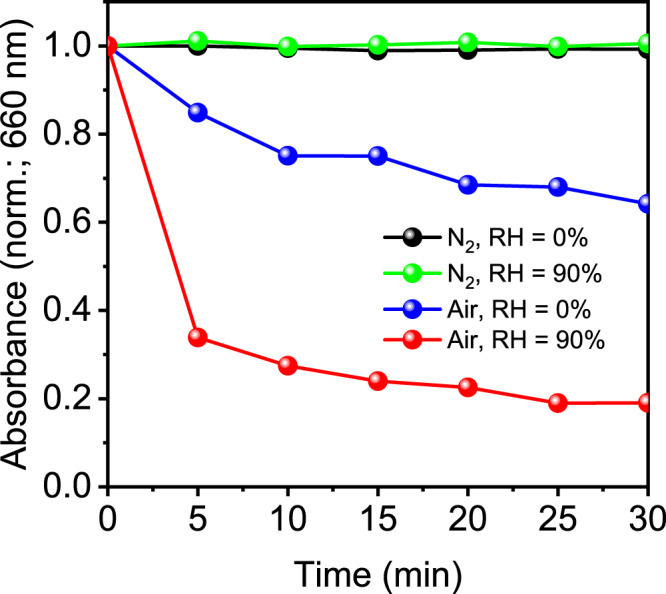
Sn perovskite degradation tests under dry/moist air/nitrogen flow. Optical degradation of (PEA)_0.2_(FA)_0.8_SnI_3_ films at 660 nm upon exposure to a flow of dry N_2_ (RH = 0%), moist N_2_ (RH = 90%), dry air (RH = 0%) and moist air (RH = 90%).

In order to obtain further evidence for our degradation mechanism presented in Fig. 1, we investigated the stability of (PEA)_0.2_(FA)_0.8_SnI_3_ films in the presence or absence of agents that trigger material decomposition, i.e., O_2_ and H_2_O. In these experiments, we exposed the perovskite films to an artificially generated flow of N_2_ or air under different relative humidity conditions (RH = 0% or RH = 90%) and tracked their optical degradation at 660 nm for a period of 30 min (see Fig. 5). We note that experimental details of these studies are provided in the Methods section. As expected, no degradation is observed when the films are exposed to dry or moist N_2_ (RH = 0% and RH = 90% respectively). The presence of H_2_O in the latter may cause hydrolysis of SnI_4_ impurities in the film to HI via Reaction 3, but O_2_ is required for the degradation cycle to progress into I_2_. Moreover, perovskite films exposed to dry air (RH = 0%) show a decay in absorbance that is consistent with the O_2_-mediated degradation of the material via Reaction 1. However, when the perovskite film is exposed to moist air (RH = 90%) optical degradation becomes much more pronounced, which we attribute to the presence of H_2_O activating the degradation cycle via Reaction 3. As such, these findings further support the proposed degradation mechanism and highlight the importance of avoiding oxygen and moisture to preserve Sn perovskite stability. We anticipate that the use of highly hydrophobic coatings in perovskite optoelectronic devices (e.g., ultrathin interlayers) will dramatically increase material resilience under ambient conditions, in accordance with the findings detailed here.

### Stabilisation of Sn perovskite films

We have shown that Sn perovskite films degrade under the oxidative action of atmospheric O_2_ and endogenously formed I_2_. These agents chemically decompose the perovskite, causing the oxidation of Sn^2+^ to Sn^4+^ to form SnI_4_. The details of the mechanism governing the degradation process provided here enable strategies to stabilise the Sn perovskite. As such, the removal of SnI_4_ and thus Sn^4+^ species from the precursor and film may help delay the perovskite decomposition in air. To further minimise the oxidation of Sn^2+^ to Sn^4+^, it is also important to consider the impact that other layers in the device (e.g., charge transport layers) may have on perovskite stability.

**Fig. 6:**
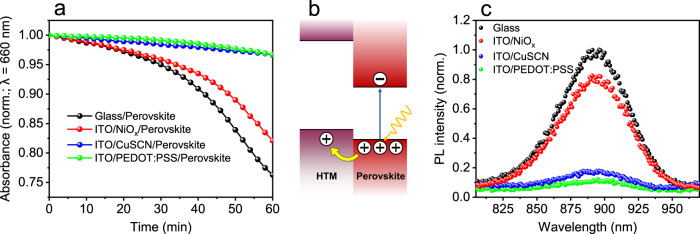
Effect of bottom hole transport layers on Sn perovskite stability. **a** Time evolution of relative absorbance at 660 nm of perovskite thin films deposited on glass and ITO/HTM substrates and exposed to ambient conditions (23 °C, 38% RH). **b** Schematic illustrating hole withdrawal from the perovskite via charge transfer to the HTM layer. **c** Steady-state PL spectra of perovskite thin films deposited on glass and ITO/HTM substrates (shown in legend). From these data, we estimate the yields of PL quenching to be 18% (ITO/NiO_*x*_/(PEA)_0.2_(FA)_0.8_SnI_3_), 82% (ITO/CuSCN/(PEA)_0.2_(FA)_0.8_SnI_3_) and 88% (ITO/PEDOT:PSS/(PEA)_0.2_(FA)_0.8_SnI_3_).

To this end, we explore the influence of the bottom, hole transport materials (HTM) on the stability of ITO/HTM/(PEA)_0.2_(FA)_0.8_SnI_3_ samples exposed to air. Specifically, NiO_*x*_, CuSCN and PEDOT:PSS were used as HTMs and glass/(PEA)_0.2_(FA)_0.8_SnI_3_ was employed as a control sample. The samples were exposed to ambient air for 60 min and UV–Visible spectroscopy was employed to monitor degradation. Figure 6a shows the absorbance at 660 nm vs. time. We note that normalised UV–Visible absorption graphs vs. wavelength and time are presented in Supplementary Fig. 16. From the data presented in Fig. 6a, we observe that the stability of the tin perovskite layer strongly depends on the substrate it is used with. For example, ITO/PEDOT:PSS/perovskite and ITO/CuSCN/perovskite samples exhibit superior stability, followed by the ITO/NiOx/perovskite sample and then glass/perovskite sample.

Next, we discuss the possible origins of this trend. NiO_*x*_, CuSCN and PEDOT:PSS have been used as HTMs in high-performance Sn perovskite solar cells^43,44,86^. We propose that NiO_x_, CuSCN and PEDOT:PSS can also accept the excess of dark holes inherently found in the Sn-based perovskite valence band that cause their unwanted p-doping (Fig. 6b). It is plausible that this effect may chemically translate as the reduction of Sn^4+^ states within the depletion region to Sn^2+^, which would delay the degradation steps described in Fig. 1 and preserve the Sn perovskite structure for longer. To test the overall ability of HTMs to withdraw holes from the perovskite, we measured the steady-state PL spectra of our samples (Fig. 6c). We observe that perovskite emission is reduced in all perovskite/HTM substrates relative to a perovskite/glass control; this is consistent with PL quenching via photoinduced hole transfer.

More direct evidence for hole transfer was obtained via time-resolved PL spectroscopy (Supplementary Fig. 17); faster charge recombination kinetics is observed in all samples containing HTM substrates, indicating efficient hole transfer from the perovskite. Taken together, the data presented in Fig. 6 suggest that the stability of the tin perovskite layer can be enhanced by improving hole transfer at the perovskite/HTM heterojunction. We note that other factors may also influence stability, such as HTM/perovskite interface passivation, chemical reactivity between HTM and perovskite and substrate-dependent point defect formation. A detailed description of the role of these issues is beyond the scope of the current manuscript and will be reported in future work.

## Discussion

Using a combination of diffraction, spectroscopy and ab initio simulation techniques, this study provides a greater understanding of the degradation mechanisms in tin perovskites, which is crucial in addressing their stability issues. The present findings illustrate the important role of SnI_4_ in the optoelectronic properties, device performance and ambient degradation of tin perovskite films based on 2D/3D (PEA)_0.2_(FA)_0.8_SnI_3_. We reveal that SnI_4_, both as a precursor impurity and as a perovskite degradation product, can rapidly evolve in ambient air to form I_2_, with the latter found to be a highly aggressive species. Specifically, we show that I_2_ causes the prompt oxidation of perovskite to form additional SnI_4_, as shown in Fig. 1.

The understanding gained from these new mechanistic insights into the degradation of the Sn-based perovskite (PEA)_0.2_(FA)_0.8_SnI_3_ enables the development of mitigation strategies aimed at reducing its sensitivity towards oxidation. We find that the choice of the bottom HTM substrate can have a significant impact on perovskite film stability. We propose that dark hole withdrawal from the perovskite can cause the chemical reduction of Sn^4+^ species to Sn^2+^, which counteracts the oxidation prompted by O_2_ and thereby delays the formation of SnI_4_ and its evolution to highly aggressive I_2_; this ultimately leads to improved stability of the tin perovskite film. In summary, this study sheds new light upon critical features of the degradation mechanism of hybrid tin iodide perovskites, paving the way towards stable and high-performance tin-based perovskite optoelectronics.

## Methods

### Materials

All chemicals were purchased from Sigma-Aldrich and used as received unless stated otherwise. Likewise, solvents were acquired from Acros Organics, unless otherwise specified, and used without further purification.

### SnI_2_ precursor purification

A SnI_2_ ampule (99.999%, trace metals basis) was opened under an N_2_-filled glovebox. The particles of the chemical were crushed in a separate vial with a metallic spatula and placed inside a covered Petri dish, which was then put onto a hot plate and heated at 250 °C for 1 h to eliminate SnI_4_ via sublimation. After the treatment, SnI_2_ is kept under dark conditions in a clean vial for future use.

### Substrate preparation

Glass microscope slides (VWR) were cut into 1.2 cm × 1.2 cm pieces and cleaned in an ultrasonic bath in three 10-min cycles (1: 4% v/v Hellmanex soap in ultrapure water; 2: acetone (VWR), 3: isopropanol (VWR)). Between every step, substrates are thoroughly rinsed with the solvent used in the previous step and then with the solvent to be used in the next step. Next, substrates are dried with a nitrogen gun and treated with UV–light/ozone for 15 min. Substrates are then used directly for perovskite/HTM deposition. ITO substrates (Psiotec, 1.2 cm × 1.2 cm, 15 Ω/cm^2^) were first rinsed with acetone and exposed to the same cleaning protocol. After UV/ozone, substrates were used immediately for HTM deposition. PEDOT:PSS (Heraeus Clevios GmbH) was filtered (0.45 µm, PTFE) and deposited via spin-coating at 5000 rpm for 30 s followed by heating at 140 °C for 20 min. 300 mg of CuSCN were dissolved in 1 mL of diethyl sulphide (Sigma Aldrich) under stirring for 5 h and filtered (0.45 µm, PTFE), followed by spin-coating deposition (4000 rpm/40 s) and heating (60 °C, 10 min). 248.8 mg of nickel acetate tetrahydrate are dissolved in 10 mL ethanol (VWR) and 60 µL ethanolamine under stirring and heating at 65 °C for 40 min and deposited via spin-coating at 4000 rpm/40 s. Substrates are then annealed at 300 °C for 1 h to obtain NiO_x_ thin films. PMMA coatings required for some perovskite layers were obtained by dissolving 10 mg of the polymer in 1 mL chlorobenzene by heating and stirring at 50 °C overnight. The solution is then applied via spin coating (2000 rpm/30 s) onto perovskite films.

### Perovskite thin film preparation

Under an inert atmosphere, a 4:1 v/v mixture of DMF and DMSO (both ultradry and bottled with molecular sieves) were filtered with 0.2 µm PTFE filters. Next, PEAI (GreatCell Solar), FAI (GreatCell Solar), SnI_2_ and SnF_2_ (99%) powders are weighed in a 0.2:0.8:1:0.1 molar ratio, respectively, to obtain 500 µL of a 0.8 M (PEA)_0.2_(FA)_0.8_SnI_3_ perovskite precursor solution by heating and stirring at 70 °C for 1 h. The solutions were then filtered (0.2 µm, PTFE) and deposited on the chosen substrate via a two-step spin-coating procedure (1: 1000 rpm/10 s; 2: 5000 rpm/30 s, 800 µL of ultradry toluene are dropped in the middle of the substrate after 12 s), followed by 70 °C annealing for 20 min.

### Sample characterisation

UV–Visible spectroscopy measurements were performed with a Shimadzu UV-2600 integrating-sphere spectrophotometer. Powder XRD patterns were acquired with a PANalytical X’Pert Pro MRD diffractometer by employing Ni-filtered Cu Kα radiation at 40 kV and 40 mA. Steady-state PL spectra were measured with a Jobin Ybon Horiba Fluoromax-3 spectrofluorometer. Time-resolved PL decays (time-correlated single-photon counting) were measured with a Horiba Deltaflex Modular Fluorescence Lifetime setup fitted with a PPD 900 detector. The excitation wavelength was chosen as 404 nm or 635 nm (whichever specified) and applied via a nanoLED (Model N-07; repetition rate: 1 MHz; pulse duration <200 ps). XPS measurements were performed on ITO/PEDOT:PSS/(PEA)_0.8_(FA)_0.2_SnI_3_ films using a Thermo K-Alpha^+^ Surface Analysis setup (photon source: Al Kα, 1486.6 eV). Transport of films from the glovebox to the measuring setup was done by using a customised transfer chamber to avoid film exposure to air. Surface scans were made under ultra-high vacuum conditions with no prior etching to avoid unwanted sample reduction. Spectral fitting and deconvolution were carried out with CasaXPS software. Possible shifts in the acquired spectra were corrected by using the C 1 s peak at 284.8 eV. TGA measurements were conducted with a Mettler Toledo Thermogravimetric analyser under an N_2_ atmosphere at a 5 °C/min heating rate. Perovskite film thickness (~250 nm) was measured with an Alpha Step Tencor D500 Surface Profilometer. SEM images were taken with an LEO 1525 Field Emission Scanning Electron Microscope used at 10 kV and fitted with an In-Lens detector. Prior to image acquisition, analysed films were unavoidably exposed to air (~1 min) and coated with 10 nm of Cr via sputtering. ^1^H-NMR measurements were taken with a 400 MHz Bruker setup, using TopSpin software.

### ^1^H-NMR sample preparation

FAI, PEAI, a fresh (PEA)_0.8_(FA)_0.2_SnI_3_ film and a (PEA)_0.8_(FA)_0.2_SnI_3_ film degraded under ambient conditions for 24 h were dissolved separately in a DMSO (VWR)/DMSO-d_6_ mixture in the glovebox and then sealed into NMR tubes and taken outside for sample measurement.

### I_2_ detection in fully aged (PEA)_0.2_(FA)_0.8_SnI_3_ films

The detection of the halogen in toluene via UV–Visible spectroscopy in perovskite films exposed to ambient air for 3 weeks was achieved by previously coating perovskite with PMMA to capture the highly volatile halogen. We note that fully degraded samples are analysed so that I_2_ does not react rapidly with perovskite as soon as it forms. In consequence, only SnI_4_ is detected in samples analysed without PMMA coating after shorter exposure times (Supplementary Fig. 15).

### Perovskite film exposure to artificial I_2_ atmospheres and characterisation

I_2_-rich atmospheres were generated by allowing I_2_ crystals to rest overnight in the glovebox at the bottom of closed vials initially containing N_2_ only, which allows I_2_ to achieve equilibrium vapour pressure. Taking *T* = 22.9 °C, we estimate the gaseous I_2_ content inside the vials to be ~0.035% (~350 ppm)^87^. Next, (PEA)_0.2_(FA)_0.8_SnI_3_ films were quickly transferred and sealed into the vials containing the I_2_ vapours and kept there for a period of 3 min, after which films were removed for their characterisation. Film degradation products were extracted with 3 mL toluene in the glovebox (films degraded with I_2_ and control fresh films kept in N_2_) or in ambient air (films degraded with I_2_ and later exposed to ambient air and films degraded under ambient air) for UV–Visible spectroscopy measurements.

### Optical degradation tests under dry/moist gas flow

In total, 1.2 cm × 0.6 cm (PEA)_0.2_(FA)_0.8_SnI_3_ films were sealed inside 1 cm × 1 cm quartz cuvettes with rubber covers under glovebox dry N_2_ environment. Cuvettes were then placed outside the glovebox and purged with constant air or N_2_ gas flow from cylinders. Prior to reaching the sample, gas is bubbled through water/glycerol solutions to control humidity (relative humidity in gas flow determined by water/glycerol ratio, as specified elsewhere^88^). Simultaneously, optical degradation of perovskite is tracked by measuring the absorbance of the sealed film every 5 min for a 30 min period.

### Device fabrication

PEDOT:PSS is deposited on ITO substrates as specified above and transferred to an N_2_-filled glovebox. A (PEA)_0.8_(FA)_0.2_SnI_3_ perovskite solution (0.8 M) made with 9:1 v/v DMF:DMSO is stirred for 1 h at 70 °C and filtered (0.2 µm, PTFE) before film deposition. The solution is applied onto PEDOT:PSS via spin-coating at 4000 rpm for 20 s. Diethyl ether is used as the antisolvent (500 µL applied at the tenth second), followed by annealing at 70 °C for 20 min. Next, a 30 mg/mL PC_60_BM (Ossila) solution in chlorobenzene is deposited at 2000 rpm for 20 s. Next, a bathocuproine (BCP) solution in isopropanol (0.5 mg/mL) is used to coat PC_60_BM at 5000 rpm for 20 s. Hundred nanometre-thick Ag electrodes are then processed via vacuum (10^−6^ mbar) thermal evaporation by using a mask leading to a 0.045 cm^2^ device active area.

### Device characterisation

J–V characteristics were measured immediately after fabrication by using simulated AM1.5 solar light (Oriel Instruments) and a Keithley 2400 source metre at a scan rate of 50 mV/s in forwarding bias. Light intensity calibration was carried out with a silicon photodiode. Devices were kept under inert conditions during the measurements by loading them in the glovebox in a homemade measuring chamber.

### Computational details

We carried out DFT^89^ calculations as implemented in the Quantum ESPRESSO package^90,91^. For the reaction energies, the Kohn–Sham wave-functions and energies are calculated with the GGA-PBEsol^92^ for electron exchange and correlation, using a plane-wave basis, with energy and charge density cutoffs of 50 and 500 Ry, respectively. Ultrasoft pseudopotentials^93^ are used to describe the core–valence interactions. The structural relaxation is performed until the force on each atom is smaller than 0.01 eV/Å. The Brillouin zone integration was sampled following the Monkhorst–Pack scheme^94^ and convergence with respect to the k-points grid was tested for each compound. For FASnI_3_, we have used a supercell of 96 atoms to allow for more degrees of freedom for a low-symmetry structure.

The reactions energies, *E*_*R*_, are calculated as the sum of total energies, *E*_*Tot*_, of the product minus the sum of total energies of the reactants (*E*_*R*_ = *n*_*i*_Σ_products_(*E*_*Tot*_(*i*)) – *n*_*i*_Σ_reactants_(*E*_*Tot*_(*i*))), where *n*_*i*_ and *E*_*tot*_ are the numbers and the total energy of the compounds and chemical species involved in the reaction. For example, the energy of reaction (1) is calculated as:

$$E_R(1)=[E_{Tot}({\mathrm{SnI}}_{4})+E_{Tot}({\mathrm{SnO}}_{2})+2E_{Tot}({\mathrm{AI}})]-[2E_{Tot}({\mathrm{ASnI}}_{3})+E_{Tot}({\mathrm{O}}_{2})].$$

In order to take into account the contribution of the zero-point energy and the entropy effects to the reaction energies, we have calculated the Gibbs free energy (*E*_*Tot*_ = *G*(*T*) = *E*_*L*_ + *E*_*0*_ + *E*_*T*_ − TS) for each reaction component at the PBE level within the harmonic approximation at 300 K as implemented in the CRYSTAL17 code^95–98^. Here *E*_*L*_ is the electronic energy corresponding to the DFT total energy at 0 K, *E*_0_ is the zero-point energy, *E*_*T*_ is the thermal contribution to the vibrational energy and TS is the entropy contribution. Effective core pseudopotentials were adopted for the heavy atoms Sn (Sn_ECP28MDF-411(51d)G), and I (I_POB_TZVP_2018) and all-electron Gaussian basis sets for N (N_pob_TZVP_2012), C (C_pob_TZVP_2012), H (H_pob_TZVP_2012) and O (O_8-411d11G_valenzano_2006) atoms. These basis sets are taken from the online library of the CRYSTAL17 code^95,96^. For the periodic systems we have used a 2 × 2 × 2 supercell for SnI_4_ (320 atoms) and FAI salt (288 atoms), 3 × 3 × 3 supercells for FASnI_3_ (324 atoms), and a 4 × 4 × 4 supercell for SnO_2_ (384). We note that the calculated reaction energies with DFT at 0 K are −4.67 eV, −0.09 eV, and 0.67 eV for reactions (1), (2), and (5), respectively, which yields similar trends as with the harmonic approximation. We also note that we have used the FASnI_3_ system for the calculation of the energetics of Reaction (1), instead of the stoichiometric compound PEA_0.2_FA_0.8_SnI_3_ studied in our experiments due to the lack of a well-defined crystal structure from XRD.

For surface calculations, we have cut the FASnI_3_ supercell structure along the crystalline plane (001) with Sn/I and FA/I terminations. The resulting slabs have symmetrically equivalent terminations to avoid large dipole effects. To maintain the bulk electronic properties, the slab thickness is chosen to be three times the above supercell of the bulk structure in the out-of-plane direction. To avoid the interaction between the repeated images, the out-of-plane direction of the box containing the slab and the vacuum layer was fixed to 50 Å. The slab without molecules was, first, fully relaxed using the generalised gradient approximation (GGA) with Perdew–Burke–Ernzerhof (PBE) formalism^99^ for the exchange-correlation. The van der Waals corrections were accounted for using the Grimme dispersion correction DFT-D3^100^. As above, ultrasoft pseudopotentials were used for describing the core–valence interactions. The energy and charge density cutoffs were set to 40 Ry and 400 Ry, respectively. The Brillouin zone integration was sampled with a 4 × 4 × 1 *k*-points grid. Dipole corrections were accounted for to eliminate dipole–dipole interactions between image supercells. For the adsorption of O_2_ or I_2_ molecules, we have considered three different configurations of the molecules on the FASnI_3_ surface, then letting them fully relax. In the case of Sn/I termination, the molecules were initially positioned at distances of 4.5 Å from the SnI_2_ surface on top of (a) I atom, (b) Sn atom, and (c) FA cation as shown in Supplementary Fig. 18. In the case of FA/I termination, two configurations were considered on top of (a) FA cation and (b) I atom, as shown in Supplementary Fig. 21. The final structures after relaxations are shown in Supplementary Figs. 19 and 20 for O_2_ and I_2_, respectively. To further examine the sensitivity of the optimised distance between the O_2_ molecules and the surface to the initial position before relaxation when the O_2_ are placed on top of FA and I (Supplementary Fig. 18a, c), we have manually placed the molecules closer to the surface than the optimised distance. After subsequent relaxation, the molecules were repelled to the optimised positions of the initial relaxation; this indicates that there is no bonding interaction between the O_2_ molecule and the FA or I at the surface.

### Reporting summary

Further information on experimental design is available in the Nature Research Reporting Summary linked to this paper.

## Supplementary information

Supplementary Information

Reporting Summary

## Data Availability

The data and computational methods that support the findings of this manuscript are available from the corresponding authors upon reasonable request.

## Code availability

The CRYSTAL and Quantum Espresso codes that were used are well established computational materials science codes. Quantum Espresso is a General Public Licensed programme and freely accessible and CRYSTAL is distributed according to specific license terms that are available from the developers.
