# Supplementary material for: Degradation mechanism of hybrid tin-based perovskite solar cells and the critical role of tin (IV) iodide
Source: Nat Commun. 2021 May 14;12:2853. doi: 10.1038/s41467-021-22864-z (PMC8121806; doi:10.1038/s41467-021-22864-z)
Supplement: Supplementary file 1 — Supplementary Information [file 41467_2021_22864_MOESM1_ESM.pdf]

Supplementary Information

Degradation Mechanism of Hybrid Tin-Based Perovskite Solar Cells and the Critical Role of Tin (IV) Iodide

Luis Lanzetta[1], Thomas Webb[1,†], Nourdine Zibouche[2,†], Xinxing Liang[1], Dong Ding[1], Ganghong Min[1], Robert J. E. Westbrook[1], Benedetta Gaggio, Thomas J. Macdonald[1], M. Saiful Islam[2]*, Saif A. Haque[1]*.

[1]Department of Chemistry and Centre for Processable Electronics, Molecular Sciences Research Hub, Imperial College London, London W12 0BZ, UK

[2]Department of Chemistry, University of Bath, Bath BA2 7AY, UK

[†]These authors contributed equally to this work

*Corresponding authors: m.s.islam@bath.ac.uk; s.a.haque@imperial.ac.uk

Supplementary Note 1: I_2-degraded perovskite films (SnI_4-rich) exposed to ambient air for further 10 minutes result in toluene extractions showing stronger absorption at ~300 and ~500 nm relative to samples exposed to I_2 vapour only (Figure 4c); this observation being consistent with the rapid evolution (< 10 min) of SnI_4 to I_2 via Reactions 3 and 4.

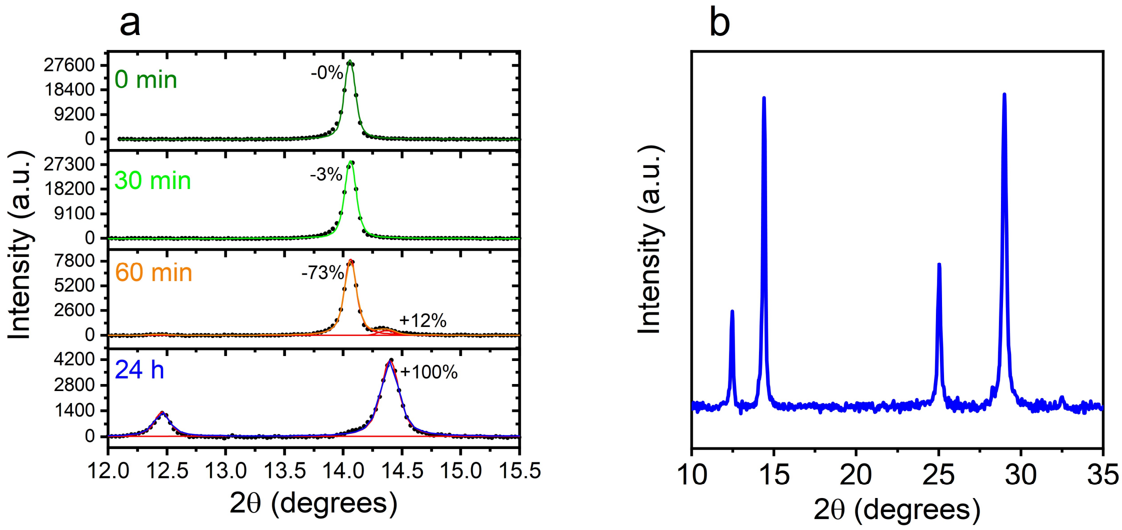

Supplementary Figure 1. a. Powder X-Ray diffraction (XRD) patterns of a $(PEA)_{0.2}(FA)_{0.8}SnI_3$ film degraded in ambient air for 0 min, 30 min, 60 min and 24 h (black dots). Patterns are deconvoluted into Voigt peak functions (colour lines: cumulative fittings; red lines: single peak functions) to estimate the percentage of perovskite signal loss (relatively to signal at 0 min) and double perovskite signal gain (normalised to signal at 24 h). **b.** Powder XRD pattern of a $(PEA)_{0.2}(FA)_{0.8}SnI_3$ thin film degraded in ambient air for 24 h. Peaks are assigned to the vacancy-ordered double perovskite derivative FA_2SnI_6. We note that the absence of SnO_2 reflections is likely owed to the highly disordered/amorphous character of this degradation product, in agreement with previous work.[1]

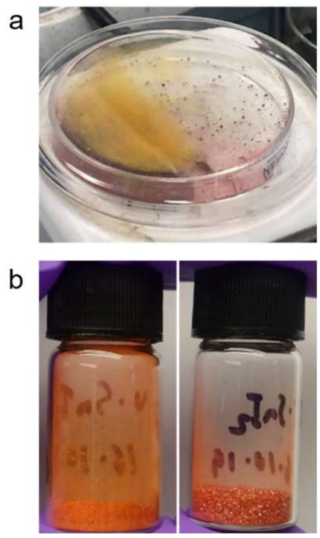

Supplementary Figure 2. SnI_2 purification heat treatment. **a.** Image of the treatment setup: commercial SnI_2 is placed inside a covered Petri dish and heated at 250ºC for 1h in a N_2-filled glovebox. SnI_4 is removed from SnI_2 via sublimation and deposited on the inner side of the lid. The purification of SnI_2 via sublimation of SnI_4 exploits the much higher volatility of the latter, being a molecular solid in contrast with ionic SnI_2. **b.** Image of vials containing SnI_2 before (left) and after (right) the purification treatment.

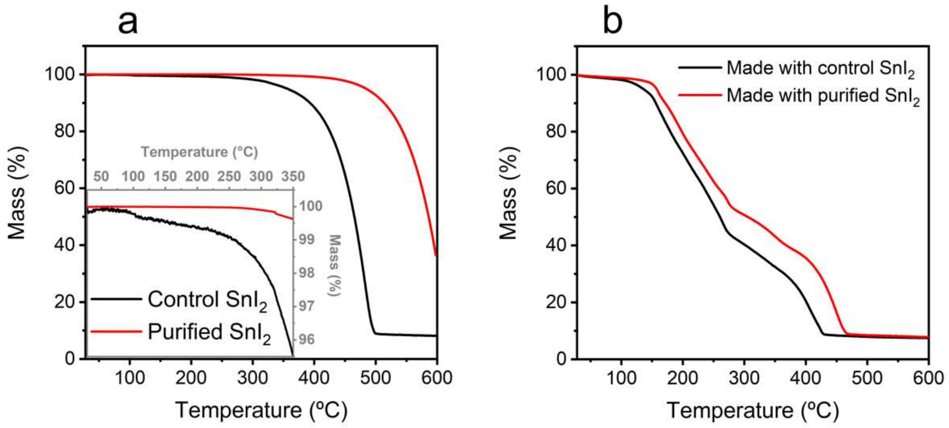

Supplementary Figure 3. Thermogravimetric analysis (TGA) of SnI_2 precursors and $(PEA)_{0.2}(FA)_{0.8}SnI_3$ perovskite registered under N_2. **a.** TGA curves of control SnI_2 and purified SnI_2. Inset: magnification of main graph at 40-350ºC temperature range. The initial decrease in mass in control SnI_2 is 0.5% approximately and starts at relatively low temperatures (~100ºC), while purified SnI_2 only exhibits a mass drop from ~300ºC attributed to SnI_2 evaporation. **b.** TGA curves of $(PEA)_{0.2}(FA)_{0.8}SnI_3$ perovskite made with control SnI_2 and purified SnI_2. The curves shows two distinctive mass losses corresponding to i) the evaporation of the organic salts and ii) the sublimation of SnI_2 at higher temperatures, in good agreement with previous reports.[2] We note that perovskite made with control SnI_2 shows mass losses at lower temperatures throughout the scan, being this difference more accentuated in the second mass loss step in consistence with SnI_2 richer in more volatile Sn^{4+} impurities.

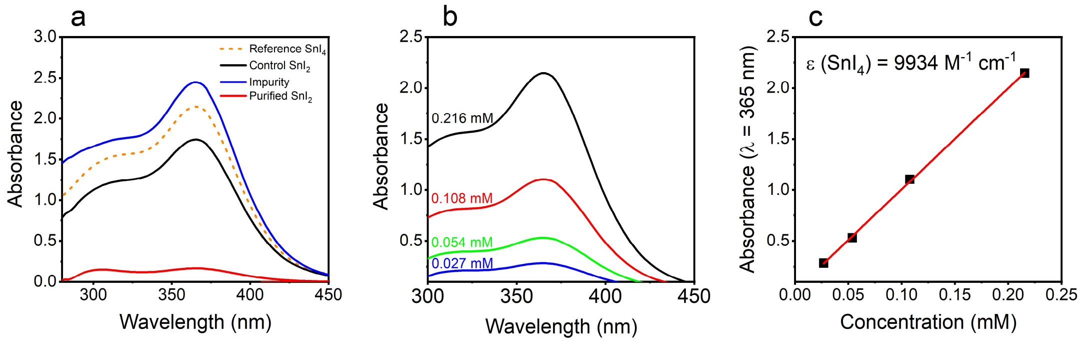

Supplementary Figure 4. a. UV-Visible absorbance spectra of impurities dissolved in ultradry toluene (SnI_4 is highly soluble in non-polar solvents like toluene, unlike SnI_2) and extracted from 0.05 g control SnI_2 (red; 3 mL toluene used), 0.05 g purified SnI_2 (black; 3 mL toluene used) and the solidified phase eliminated from control SnI_2 via sublimation (blue). These absorption spectra match the one recorded for a 0.216 mM SnI_4 reference solution in toluene (dashed orange line), allowing to identify the impurity as SnI_4. **b.** UV-Visible absorbance spectra of reference SnI_4 solutions in toluene at different concentrations. **c.** Absorbance (λ = 365 nm) vs concentration plot obtained from data in Supplementary Figure 4b and its linear fitting ($\varepsilon(SnI_4)$ = 9934 M^{-1} cm^{-1}). We obtain the mass percentage of SnI_4 in the precursor before and after the heat treatment by applying the Beer-Lambert Law ($A = \varepsilon cl$; l = 1 cm) to calculate the concentration of SnI_4 in the toluene extractions (Supplementary Figure 4a) from their absorbance at 365 nm. Control SnI_2 contains 0.65% SnI_4 in mass, while only 0.06% SnI_4 is found in purified SnI_2.

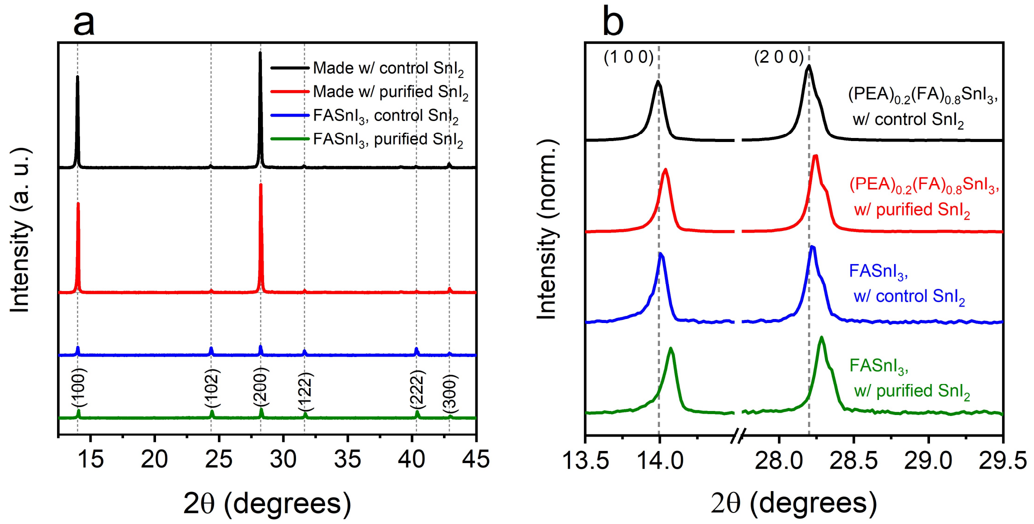

Supplementary Figure 5. a. Powder XRD patterns of glass/$(PEA)_{0.2}(FA)_{0.8}SnI_3$ and $FASnI_3$ thin films made with control/purified SnI_2. The indexed diffraction peaks reveal that perovskites possess an orthorhombic crystal structure (Amm2 space group), consistent with the α-phase of $FASnI_3$.[3] In contrast with archetypal 3D $FASnI_3$ film, peaks in patterns of $(PEA)_{0.2}(FA)_{0.8}SnI_3$ films provide much higher diffraction intensity, indicating that the addition of PEAI increases the crystallinity of the final perovskite. Furthermore, this also causes (h00) reflections in the diffractogram to clearly become more prominent (particularly (100) and (200)), which suggests that perovskite crystals preferentially grow with their {h00} planes oriented parallel to the substrate.[4,5] Diffraction patterns of $(PEA)_{0.2}(FA)_{0.8}SnI_3$ do not present significant changes when the control or purified SnI_2 is employed, confirming that the perovskite crystalline structure is not altered as a consequence of the purification of the precursor. **b.** Magnified X-ray diffraction patterns of (100) and (200) main reflections acquired from $(PEA)_{0.2}(FA)_{0.8}SnI_3$ and $FASnI_3$ films made with control/purified SnI_2. Peaks from perovskites made with purified SnI_2 are shifted to higher diffraction angles, indicative of smaller lattice parameters. We suggest that reducing the incorporation of Sn^{4+} into perovskite may result in less Sn^{2+}/I^- vacancies in the lattice and therefore more compact unit cells.

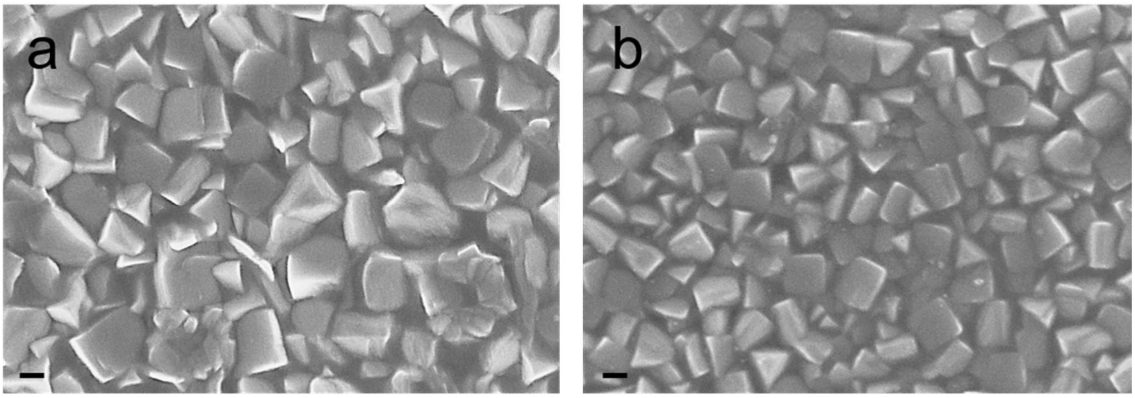

Supplementary Figure 6. Top-view Scanning Electron Microscopy (SEM) images of $(PEA)_{0.2}(FA)_{0.8}SnI_3$ perovskite thin films deposited on PEDOT:PSS. Scale bar: 200 nm. **a.** Film made with control SnI_2. **b.** Film made with purified SnI_2. SEM images reveal submicron-sized quasi-cubic perovskite grains, being these slightly smaller in samples made with purified SnI_2.

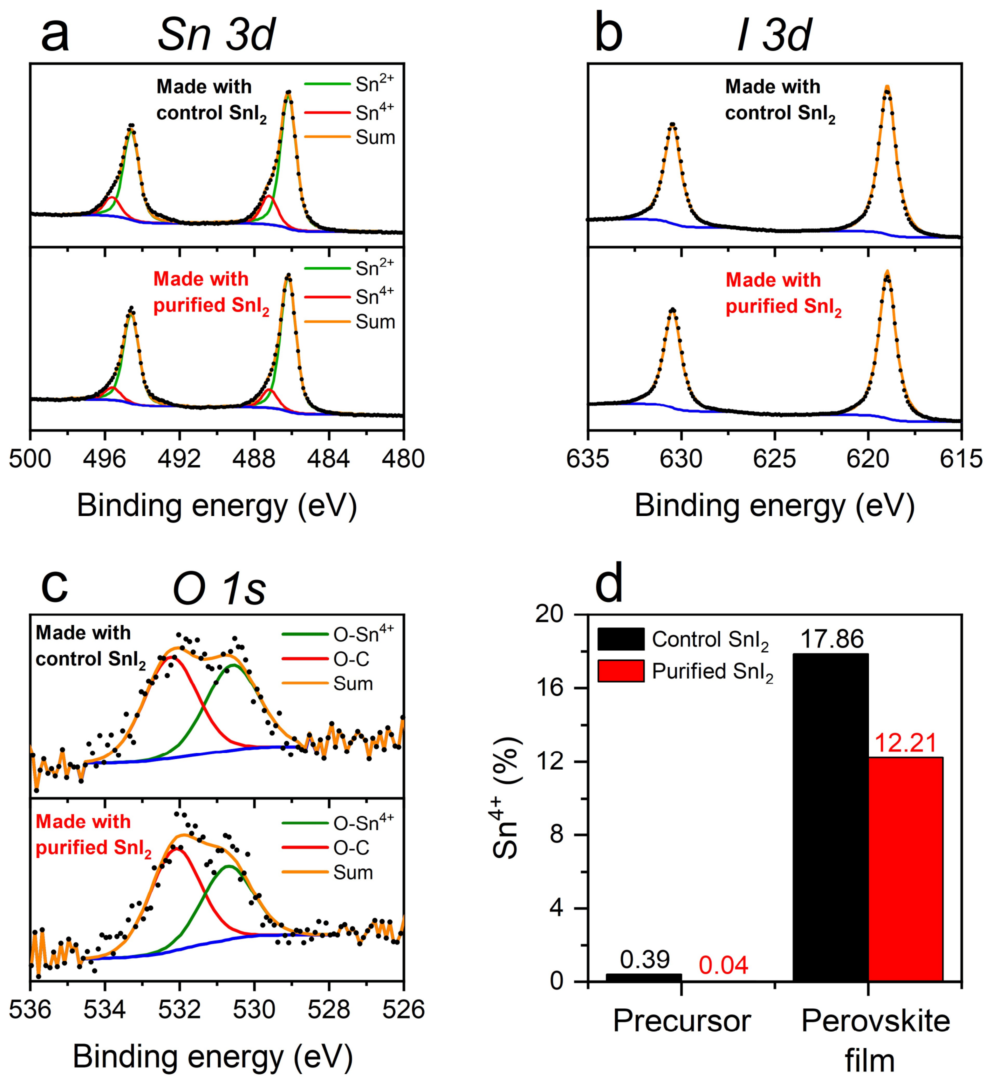

Supplementary Figure 7. Surface X-Ray Photoelectron Spectroscopy (XPS) spectra of ITO/PEDOT:PSS/$(PEA)_{0.2}(FA)_{0.8}SnI_3$ films made with control/purified SnI_2 and their deconvolution. Further details on species assignation are provided in Supplementary Table 1. **a.** Sn $3d_{5/2}$ and Sn $3d_{3/2}$ XPS peaks of perovskite films. We deconvolute the XPS spectra into two regions assigned to Sn^{2+} in perovskite (~486.18 eV, ~494.59 eV) and a combination of Sn^{4+} states (SnO_2 + perovskite Sn^{4+} centres; ~487.21 eV, ~495.62 eV). The total contribution (peak area percentage) of the Sn^{4+} component decreases from 17.86% to 12.21% when using purified SnI_2 instead of control SnI_2, verifying that using Sn^{4+}-poorer precursors leads to fewer Sn^{4+} states in the perovskite. **b.** I $3d_{5/2}$ and I $3d_{3/2}$ XPS peaks of perovskite films. Spectra are fitted to a single component assigned to iodide in the perovskite structure. **c.** O 1*s* spectra of perovskite films. Spectra are deconvoluted into two regions assigned to SnO_2 (O-Sn^{4+}; ~530.66 eV) and adventitious organic contaminants (O-C; 532.18 eV). **d.** Sn^{4+} content (i.e. percentage of Sn atoms with oxidation state IV) in SnI_2 precursors (measured with UV-Visible spectroscopy; Beer-Lambert Law) and $(PEA)_{0.2}(FA)_{0.8}SnI_3$ films (measured with XPS). We find that the percentage of Sn^{4+} states in the perovskite films is considerably higher compared to the values obtained in the SnI_2 precursors, suggesting that Sn^{2+} undergoes oxidation in the intermediate steps of film processing/storage prior to measurement.

Precursor used		Sn^{2+}	Sn^{4+}	I^-	$O\text{-}Sn^{4+}$
**Control SnI_2**	**Area/(RSF*T*MFP)**	2612.310	567.974	8058.600	356.603
	**Atomic ratio vs Sn^{2+}**	1.000	0.217	3.085	0.137
**Purified SnI_2**	**Area/(RSF*T*MFP)**	2747.850	382.075	8648.270	367.869
	**Atomic ratio vs Sn^{2+}**	1.000	0.139	3.147	0.134

Supplementary Table 1. Corrected areas and atomic ratios (vs Sn^{2+}) obtained from XPS components in Supplementary Figures 7a, 7b and 7c. We observe that the $O\text{-}Sn^{4+}$ ratios are comparable in both samples (0.137 vs 0.134), indicating a similar SnO_2 concentration at the surface in both cases. Assuming 1:2 stoichiometry, the Sn^{4+} ratios corresponding to SnO_2 are estimated as 0.069 and 0.067 for films made with control and purified SnI_2, respectively. The remaining Sn^{4+} states (control SnI_2: 0.217-0.069 = 0.148; purified SnI_2: 0.139-0.067 = 0.072) are assigned to Sn^{4+} centres in the perovskite structure. Hence, the main effect of using purified precursors is a decrease in Sn^{4+} centres in the perovskite. We consider any major contribution from SnI_4 or FA_2SnI_6 to the Sn^{4+} component unlikely due to i) SnI_4 being highly volatile and rapidly evaporating from the film at vacuum levels used in XPS (10^{-6} mbar or lower) and ii) the I^- ratios being close to 3 in consistence with the perovskite stoichiometry (I^- ratios expected to be significantly higher if Sn^{4+} components were caused by SnI_4 or FA_2SnI_6).

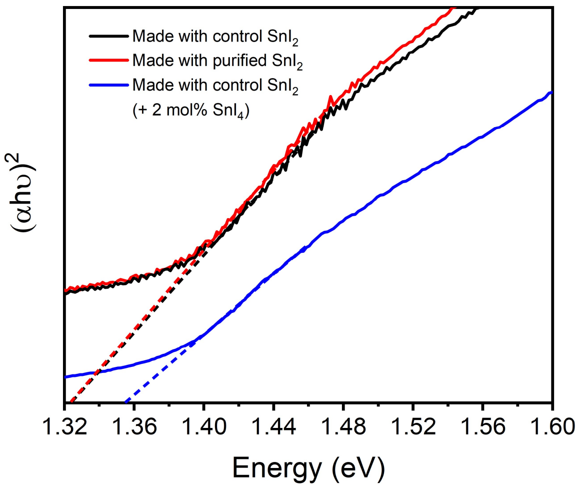

Supplementary Figure 8. Tauc plots of $(PEA)_{0.2}(FA)_{0.8}SnI_3$ perovskite films made with control SnI_2 (black line), purified SnI_2 (red line) and control SnI_2 + 2 mol% SnI_4. The resulting bandgaps are 1.32 eV, 1.32 eV and 1.35 eV, respectively.

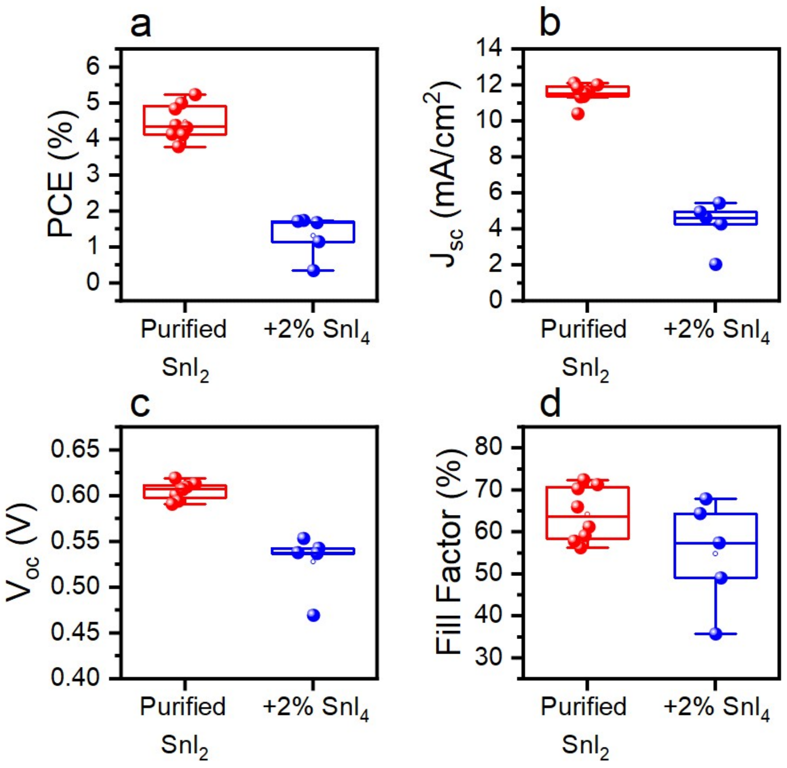

Supplementary Figure 9. a. PCE, **b.** J_{sc}, **c.** V_{oc} and **d.** Fill Factor statistics of cells made with purified SnI_2 (8 devices) and with control SnI_2 + 2 mol% SnI_4 (5 devices). Box range: 25th-75th percentiles; centre line: median; whiskers: 1.5x interquartile range.

Precursor Type	PCE(%)	V_{oc} (V)	J_{sc}	FF (%)
**Purified SnI_2**	4.47 ± 0.50 (5.23)	0.61 ± 0.01 (0.61)	11.52 ± 0.54 (12.00)	64.27 ± 6.50 (71.19)
**Control SnI_2 (+ 2% SnI_4)**	1.32 ± 0.60 (1.73)	0.53 ± 0.03 (0.55)	4.26 ± 1.31 (4.61)	54.89 ± 12.94 (67.90)

Supplementary Table 2. Mean values for solar cell PCE, J_{sc}, V_{oc} and Fill Factor and their standard deviations. Values in parentheses correspond to parameters of champion devices.

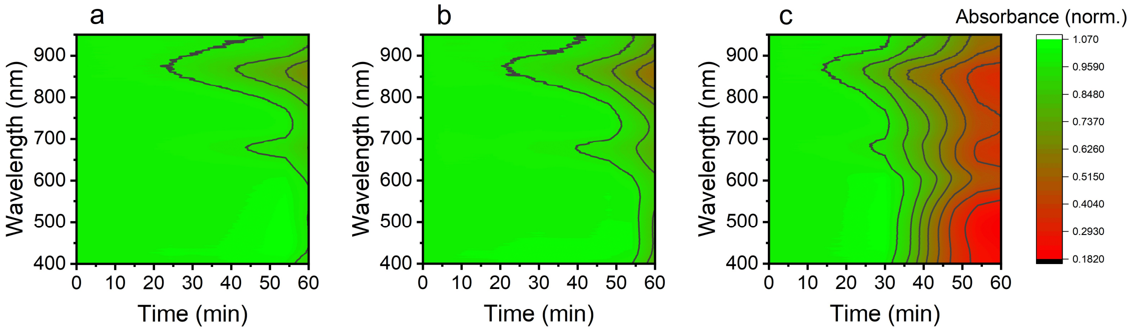

Supplementary Figure 10. Contour graphs representing $(PEA)_{0.2}(FA)_{0.8}SnI_3$ normalised absorbance (Abs(t)/Abs(0)) as a function of time and wavelength in films made with **a.** purified SnI_2, **b.** control SnI_2 and **c.** control SnI_2 + 2 mol% SnI_4.

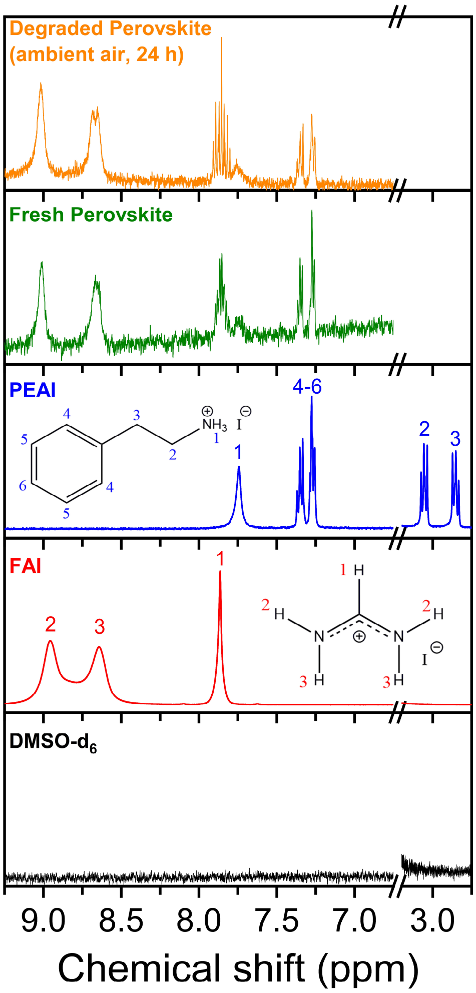

Supplementary Figure 11. Downfield proton nuclear magnetic resonance (^{1}H-NMR) spectra of DMSO-d_6, FAI, PEAI, fresh $(PEA)_{0.2}(FA)_{0.8}SnI_3$ and $(PEA)_{0.2}(FA)_{0.8}SnI_3$ aged for 24h in ambient air. Peaks in FAI ad PEAI spectra are assigned to protons in their respective molecules (see inset). The observed increase in multiplicity in degraded perovskite peaks assigned to FAI is attributed to the inhibition of the dynamic proton exchange between water and FA^+ upon H_2O protonation.[6] This is attributed to the presence of SnI_4 and its reaction with H_2O to form HI.

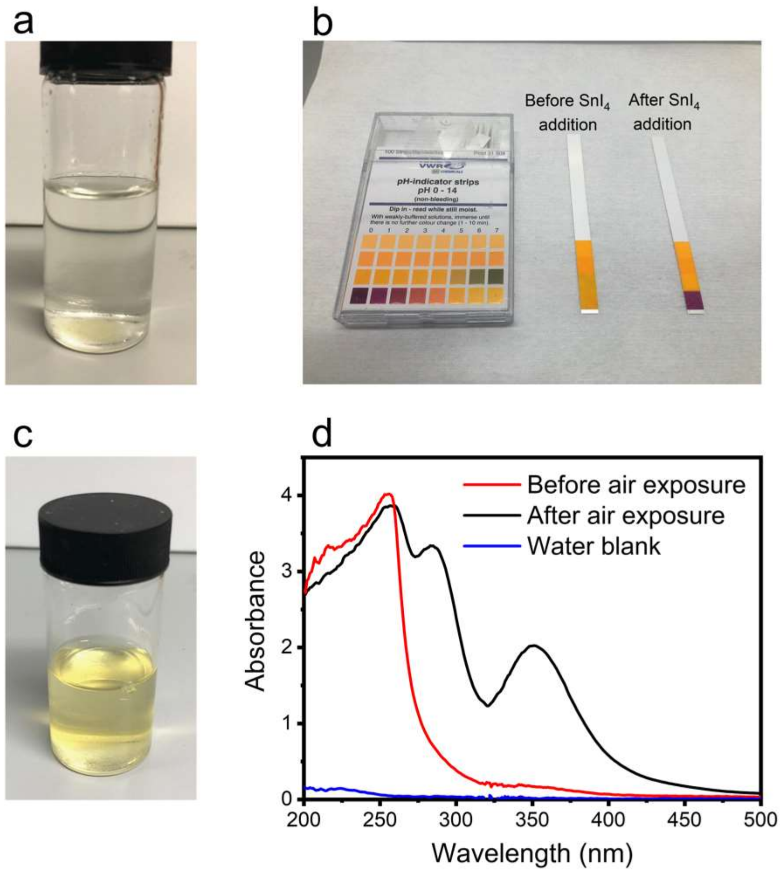

Supplementary Figure 12. a. Image of a vial filled with degassed deionized water after the addition of SnI_4. The white precipitate at the bottom corresponds to the formation of SnO_2 via Reaction 3 in the main paper. This experiment was carried out under inert N_2 atmosphere to rule out the involvement of O_2 in the reaction. **b.** pH test of deionized water vial before and after SnI_4 addition under N_2 atmosphere. The acidification of water upon SnI_4 addition is indicative of the formation of HI via hydrolysis (Reaction 3). **c.** Image of the SnI_4-treated vial after 4h exposure to ambient air. The change in colour from transparent to yellow/light brown indicates the presence of I_3^- species. **d.** UV-Visible absorbance spectra of water before SnI_4 addition (blank), after SnI_4 addition/before air exposure and after SnI_4 addition/after 4h air exposure. The feature at ~ 250 nm is assigned to UV absorption by SnO_2 crystals (formed via Reaction 3). After air exposure, HI in solution is oxidized to I_2, which combines with remaining I^- anions to give I_3^- (peaks at ~285 and ~350 nm).

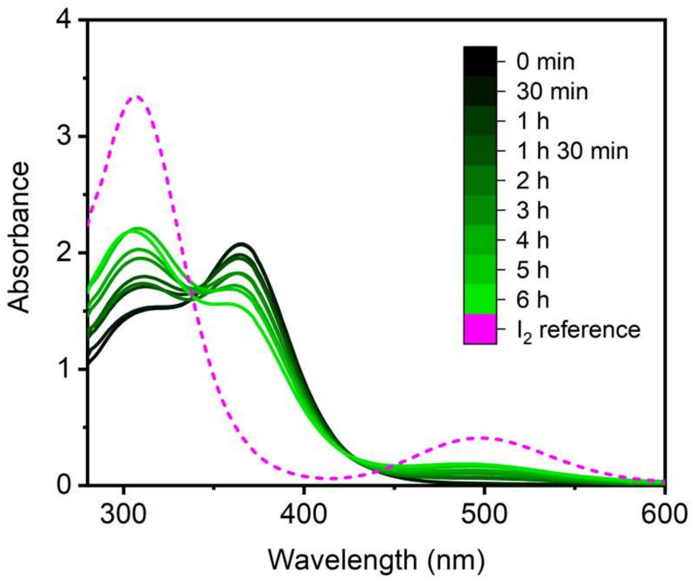

Supplementary Figure 13. Absorbance time evolution of a SnI_4 reference solution in toluene left in air for 6 hours and spectrum of an I_2 reference solution in toluene. The decrease of the absorption band at ~360 nm, the rise of spectral features at ~300 nm and ~500 nm and a colour change from orange to purple confirm the evolution of SnI_4 to I_2 in solution. Spectral changes over time are detected after only 30 min of ambient air exposure, suggesting the degradation of SnI_4 to I_2 is a relatively fast process.

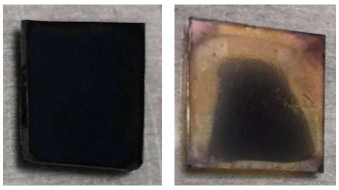

Supplementary Figure 14. Images of $(PEA)_{0.2}(FA)_{0.8}SnI_3$ perovskite thin films made with purified SnI_2 before degradation (left) and after being exposed to I_2 vapours in a N_2-filled glovebox for 3 minutes (right).

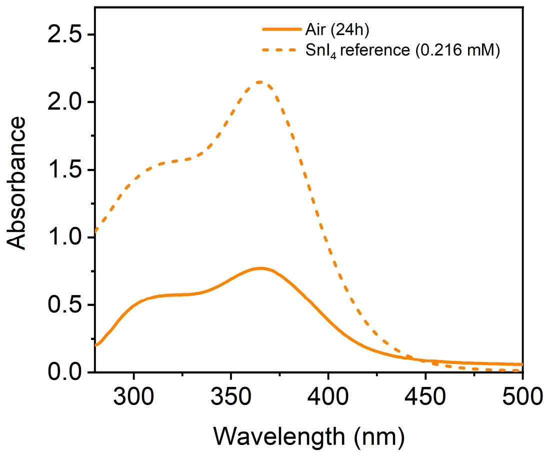

Supplementary Figure 15. UV-Visible spectrum of the degradation products extracted in toluene from a PMMA-free $(PEA)_{0.2}(FA)_{0.8}SnI_3$ thin film aged in ambient air for 24 h. The spectrum fully matches that of the SnI_4 reference solution (dashed line) and no features corresponding to I_2 are detected.

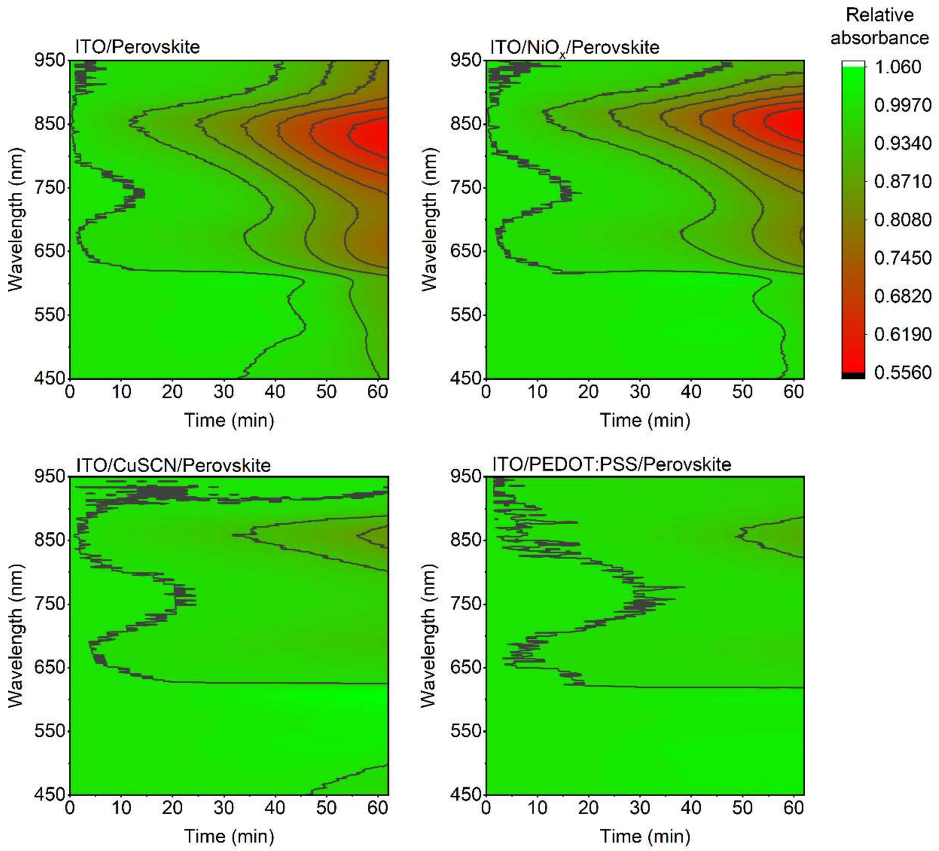

Supplementary Figure 16. Contour graphs showing the absorbance decay of $(PEA)_{0.2}(FA)_{0.8}SnI_3$ perovskite deposited on ITO and ITO/HTM substrates upon 1-hour exposure to ambient conditions (23°C, 38% RH).

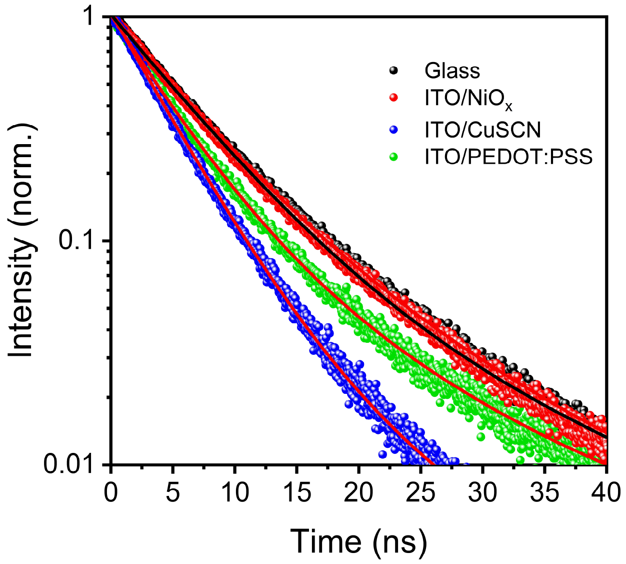

Supplementary Figure 17. Time-resolved PL decays of $(PEA)_{0.2}(FA)_{0.8}SnI_3$ films deposited on glass and ITO/HTM substrates acquired at 875 nm with 635 nm excitation and their biexponential fitting curves. Average τ_f values are 7.38 ns (glass/Perovskite), 7.31 ns (ITO/NiO_x/Perovskite), 4.71 ns (ITO/CuSCN/Perovskite) and 5.79 ns (ITO/PEDOT:PSS/Perovskite).

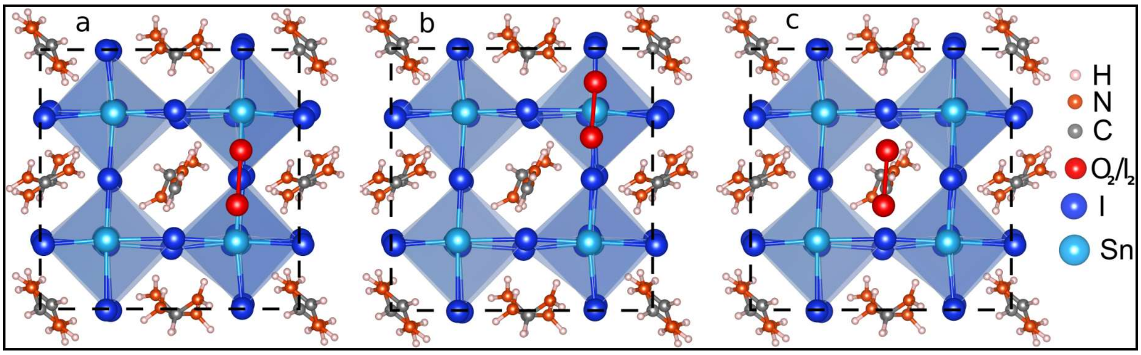

Supplementary Figure 18. Top view of the initial configurations of O_2 or I_2 molecules (red) on the (001) $FASnI_3$ surface with SnI_2 termination considered in this study. **a**. On top of the I atom, **b**. on top of the Sn and **c.** on the of the FA molecule of the $FASnI_3$ slab.

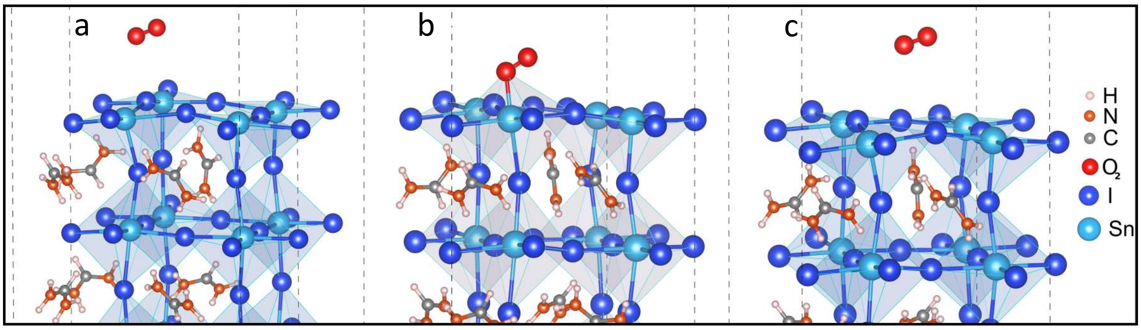

Supplementary Figure 19. Side view of the final configurations of O_2 molecule (red) on the (001) $FASnI_3$ surface with SnI_2 termination after relaxation starting **a**. on top of the I atom, **b**. on top of the Sn and **c**. on the of the FA molecule of the $FASnI_3$ slab.

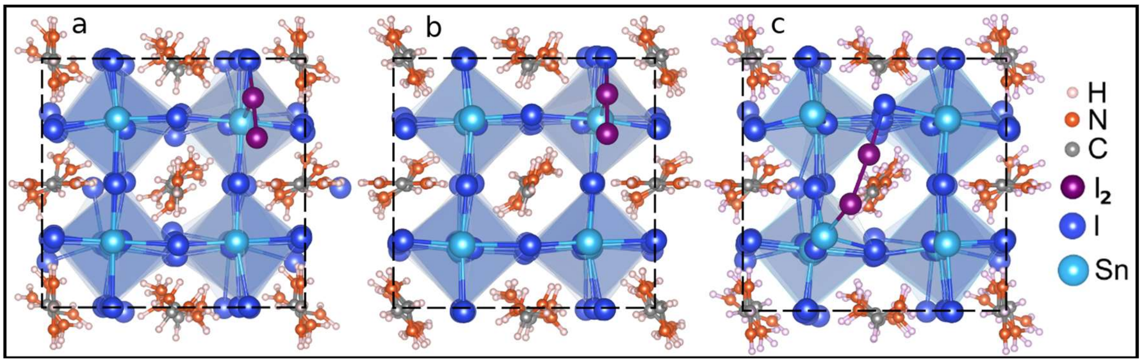

Supplementary Figure 20. Top view of the final configurations of I_2 molecule (red) on the (001) $FASnI_3$ surface with SnI_2 termination after relaxation starting **a**. on top of the I atom, **b**. on top of the Sn, and **c**. on the of the FA molecule of the $FASnI_3$ slab.

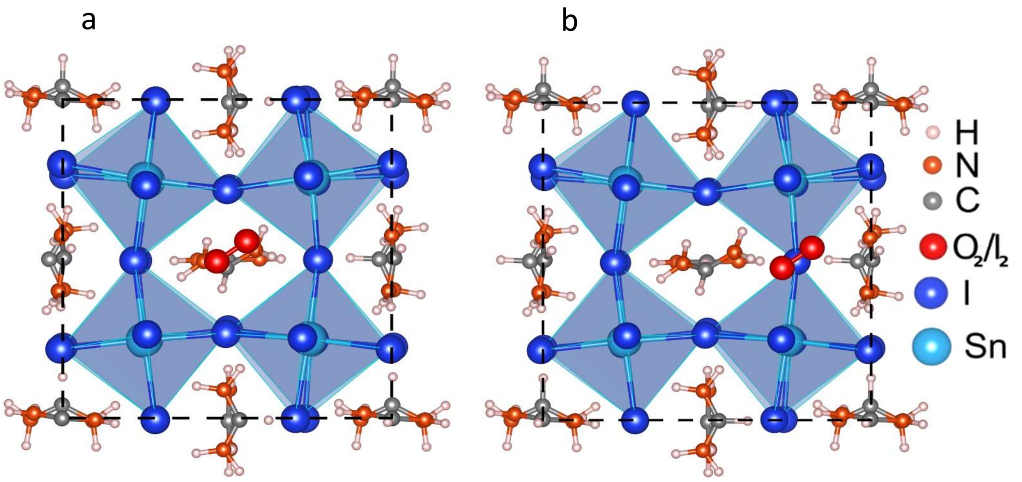

Supplementary Figure 21. Top view of the initial configurations of O_2 or I_2 molecules (red) on the (001) $FASnI_3$ surface with FAI termination considered in this study. **a**. On top of the FA molecule of the $FASnI_3$ slab. **b**. On top of the iodine atom of the $FASnI_3$ slab.

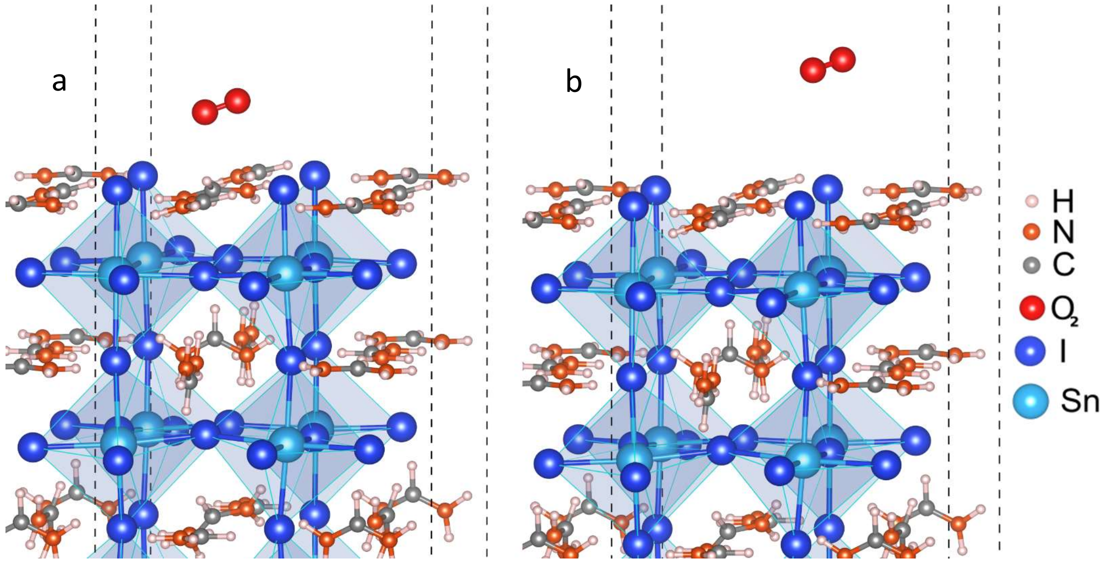

Supplementary Figure 22. Side view of the final configurations of O_2 molecule (red) on the (001) $FASnI_3$ surface with FAI termination after relaxation starting **a**. on top of the FA molecule or **b**. on top of the iodine atom of the $FASnI_3$ slab.

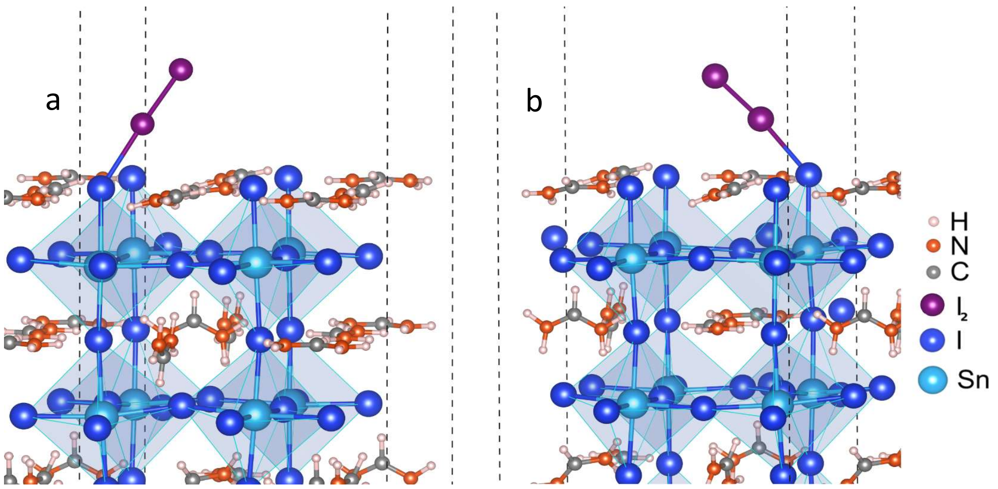

Supplementary Figure 23. Side view of the final configurations of I_2 molecule (purple colour) on the (001) $FASnI_3$ surface with FAI termination after relaxation starting **a**. on top of the FA molecule or **b**. on top of the iodine atom of the $FASnI_3$ slab.

Supplementary References

1. Kubicki, D. J. *et al.* Local Structure and Dynamics in Methylammonium, Formamidinium, and Cesium Tin(II) Mixed-Halide Perovskites from ^{119}Sn Solid-State NMR. *J. Am. Chem. Soc.* **142**, 7813–7826 (2020).
2. Leijtens, T., Prasanna, R., Gold-Parker, A., Toney, M. F. & McGehee, M. D. Mechanism of Tin Oxidation and Stabilization by Lead Substitution in Tin Halide Perovskites. *ACS Energy Lett.* **2**, 2159–2165 (2017).
3. Stoumpos, C. C., Malliakas, C. D. & Kanatzidis, M. G. Semiconducting Tin and Lead Iodide Perovskites with Organic Cations: Phase Transitions, High Mobilities, and Near-Infrared Photoluminescent Properties. *Inorg. Chem.* **52**, 9019–38 (2013).
4. Liao, Y. *et al.* Highly Oriented Low-Dimensional Tin Halide Perovskites with Enhanced Stability and Photovoltaic Performance. *J. Am. Chem. Soc.* **139**, 6693–6699 (2017).
5. Rath, T. *et al.* Photovoltaic properties of a triple cation methylammonium/formamidinium/phenylethylammonium tin iodide perovskite. *J. Mater. Chem. A* **7**, 9523–9529 (2019).
6. Van Gompel, W. T. M. *et al.* Degradation of the Formamidinium Cation and the Quantification of the Formamidinium−Methylammonium Ratio in Lead Iodide Hybrid Perovskites by Nuclear Magnetic Resonance Spectroscopy. *J. Phys. Chem. C* **122**, 4117–4124 (2018).
