# Supplementary material for: Degradation mechanism of hybrid tin-based perovskite solar cells and the critical role of tin (IV) iodide
Source: Nat Commun. 2021 May 14;12:2853. doi: 10.1038/s41467-021-22864-z (PMC8121806; doi:10.1038/s41467-021-22864-z)
Supplement: Supplementary file 2 — Reporting Summary [file 41467_2021_22864_MOESM2_ESM.pdf]

naturereresearch

Corresponding author(s): Saif A. Haque, M. Saiful Islam

Solar Cells Reporting Summary

Nature Research wishes to improve the reproducibility of the work that we publish. This form is intended for publication with all accepted papers reporting the characterization of photovoltaic devices and provides structure for consistency and transparency in reporting. Some list items might not apply to an individual manuscript, but all fields must be completed for clarity.

For further information on Nature Research policies, including our data availability policy, see Authors & Referees.

Experimental design

Please check: are the following details reported in the manuscript?

1. Dimensions

Item	Yes	No	Comment
Area of the tested solar cells	☒	☐	Methods section
Method used to determine the device area	☒	☐	Methods section

2. Current-voltage characterization

Item	Yes	No	Comment
Current density-voltage (J-V) plots in both forward and backward direction	☐	☒	Not relevant to our study
Voltage scan conditions<br>*For instance: scan direction, speed, dwell times*	☒	☐	Methods section
Test environment<br>*For instance: characterization temperature, in air or in glove box*	☒	☐	Methods section
Protocol for preconditioning of the device before its characterization	☒	☐	Methods section
Stability of the J-V characteristic<br>*Verified with time evolution of the maximum power point or with the photocurrent at maximum power point; see ref. 7 for details.*	☐	☒	Not relevant to our study

3. Hysteresis or any other unusual behaviour

Item	Yes	No	Comment
Description of the unusual behaviour observed during the characterization	☐	☒	Not relevant to our study
Related experimental data	☐	☒	Not relevant to our study

4. Efficiency

Item	Yes	No	Comment
External quantum efficiency (EQE) or incident photons to current efficiency (IPCE)	☐	☒	Not relevant to our study
A comparison between the integrated response under the standard reference spectrum and the response measure under the simulator	☐	☒	Not relevant to our study
For tandem solar cells, the bias illumination and bias voltage used for each subcell	☐	☒	Not relevant to our study

5. Calibration

Item	Yes	No	Comment
Light source and reference cell or sensor used for the characterization	☒	☐	Methods section
Confirmation that the reference cell was calibrated and certified	☐	☒	Not relevant to our study

Calculation of spectral mismatch between the reference cell and the devices under test

- [ ] Yes
- [x] No

Not relevant to our study

6. Mask/aperture

Size of the mask/aperture used during testing

- [ ] Yes
- [x] No

Not relevant to our study

Variation of the measured short-circuit current density with the mask/aperture area

- [ ] Yes
- [x] No

Not relevant to our study

7. Performance certification

Identity of the independent certification laboratory that confirmed the photovoltaic performance

- [ ] Yes
- [x] No

Not relevant to our study

A copy of any certificate(s)
Provide in Supplementary Information

- [ ] Yes
- [x] No

Not relevant to our study

8. Statistics

Number of solar cells tested

- [x] Yes
- [ ] No

Figure S9, Supplementary Information

Statistical analysis of the device performance

- [x] Yes
- [ ] No

Figure S9 and Table S2, Supplementary Information

9. Long-term stability analysis

Type of analysis, bias conditions and environmental conditions
For instance: illumination type, temperature, atmosphere humidity, encapsulation method, preconditioning temperature

- [ ] Yes
- [x] No

Not relevant to our study
